# Supplementary material for: Protocol for the Work Engagement and Well-being Study (SWELL): a randomised controlled feasibility trial evaluating the effects of mindfulness versus light physical exercise at work
Source: BMJ Open. 2022 Apr 11;12(4):e050951. doi: 10.1136/bmjopen-2021-050951 (PMC9006806; doi:10.1136/bmjopen-2021-050951)
Supplement: Author's manuscript [file bmjopen-2021-050951.draft_revisions.pdf]

BMJ Open

BMJ Open is committed to open peer review. As part of this commitment we make the peer review history of every article we publish publicly available.

When an article is published we post the peer reviewers' comments and the authors' responses online. We also post the versions of the paper that were used during peer review. These are the versions that the peer review comments apply to.

The versions of the paper that follow are the versions that were submitted during the peer review process. They are not the versions of record or the final published versions. They should not be cited or distributed as the published version of this manuscript.

BMJ Open is an open access journal and the full, final, typeset and author-corrected version of record of the manuscript is available on our site with no access controls, subscription charges or pay-per-view fees (http://bmjopen.bmj.com).

If you have any questions on BMJ Open's open peer review process please email info.bmjopen@bmj.com

BMJ Open

# Protocol for the Work Engagement and Well-being Study (SWELL): A randomised controlled feasibility trial evaluating the effects of mindfulness versus light physical exercise at work


| | |
|---|---|
| Journal: | *BMJ Open* |
| Manuscript ID | bmjopen-2021-050951 |
| Article Type: | Protocol |
| Date Submitted by the Author: | 23-Jun-2021 |


| | |
|---|---|
| Complete List of Authors: | Vainre, Maris; University of Cambridge<br>Galante, Julieta; University of Cambridge, Department of Psychiatry<br>Watson, Peter; University of Cambridge, MRC Cognition and Brain Sciences Unit<br>Dalgleish, Tim; Medical Research Council Cognition and Brain Sciences Unit<br>Hitchcock, Caitlin; University of Cambridge, MRC Cognition and Brain Sciences Unit |


| | |
|---|---|
| Keywords: | PUBLIC HEALTH, PREVENTIVE MEDICINE, OCCUPATIONAL & INDUSTRIAL MEDICINE |


SCHOLARONE™
Manuscripts

# Protocol for the Work Engagement and Well-being Study (SWELL): A randomised controlled feasibility trial evaluating the effects of mindfulness versus light physical exercise at work

Ms Maris Vainre, maris.vainre@mrc-cbu.cam.ac.uk, MRC Cognition and Brain Sciences Unit, University of Cambridge, Cambridge, UK

Dr Julieta Galante, Department of Psychiatry, University of Cambridge, Cambridge, UK

Dr Peter Watson, MRC Cognition and Brain Sciences Unit, University of Cambridge, Cambridge, UK

Prof Tim Dalgleish, MRC Cognition and Brain Sciences Unit, University of Cambridge, Cambridge, UK, Cambridgeshire and Peterborough NHS Foundation Trust, Cambridgeshire and Peterborough, UK

Dr Caitlin Hitchcock, MRC Cognition and Brain Sciences Unit, University of Cambridge, Cambridge, UK

Word count: 4000 excluding title page, abstract, references and references to tables and images.

Keywords: mindfulness, work, well-being, productivity, randomised controlled trial

For peer review only

# ABSTRACT

## Introduction

Mental ill health is a major cause of disability. Workplaces are attractive for preventative interventions since most adults work while employers are interested in improving employees' well-being and productivity. Mindfulness-based programmes are increasingly in occupational settings. However, there is inconsistent evidence whether mindfulness interventions improve work performance and how effective mindfulness-based programmes are, compared to other interventions, in preventing mental ill health.

## Methods and analysis

In this online randomised controlled feasibility trial, an anticipated 240 employees will be randomised to either a 4-week light physical exercise course or a mindfulness course of the same duration (1:1 allocation). The primary outcome is work performance, measured using the Work Role Functioning Questionnaire. We aim to evaluate the acceptability, feasibility, and procedural uncertainties of a randomised controlled trial in a workplace, calculate an effect size estimate to inform power calculations for a larger trial, and explore whether improved executive function and/or enhanced mental health could be potential mechanisms underlying the effect of mindfulness on work performance. Outcomes will be collected at baseline, post-intervention and 12-week follow-up.

## Ethics and dissemination

Approval has been obtained from Cambridge Psychology Research Ethics Committee (PRE.2020.072). Results will be published in peer-reviewed journals. A lay summary will be disseminated to a wider audience including participating employers.

## Registration details

Clinicaltrials.gov: NCT04631302


# ARTICLE SUMMARY

Strengths and limitations of this study:

- A randomised trial to lay the foundations to investigate the mechanisms of mindfulness intervention underlying effects on work performance.
- The study employs a range of outcome measures, including self-reported measures and cognitive functioning tasks.

For peer review only

- This study is not powered to detect significant effects, but rather to estimate effect size to inform design of a larger later-stage trial.
- Several feasibility outcomes will be collected to inform a later-stage trial.

For peer review only

# INTRODUCTION

## Background and rationale

Mental illness is a major cause of disability worldwide[1]. Much of the adult population is employed and spends 28% of their waking hours doing paid work[2,3]. The occupational environment is therefore an opportune location for preventative mental health interventions.

Poor mental health is responsible for 44% of work-related episodes of ill health[4] and according to conservative estimates, is thought to cost the United Kingdom's (UK) economy £45 billion annually[5] or 2% of UK's Gross Domestic Product. To reduce this burden, a growing number of employers provide programmes to improve well-being and work performance.

Mindfulness-based programmes (MBPs) are increasingly popular in occupational settings. Mindfulness is typically defined as "the awareness that emerges through paying attention on purpose, in the present moment, and nonjudgmentally to the unfolding of experience moment by moment"[6]. Practicing such awareness is linked to reduction in symptoms of anxiety, depression, and stress in community populations[7,8]. There is also evidence that mindfulness training could improve overall well-being[8,9], life satisfaction[10], and quality of life[7].

Mindfulness practice may yield workplace benefits beyond emotional well-being. It has been proposed that mindfulness improves work performance[11] and reduces the negative effects of multitasking[12]. Yet, there is little evidence to support these claims. A recent meta-analysis concluded that work performance was rarely assessed in trials investigating the outcomes of MBPs. When work performance was measured, wide-ranging operational definitions were used: e.g., engagement[13–17], motivation[14], absenteeism[18] and presenteeism[16–18], rate of errors[19] and burnout[20]. Thus, estimating an overall effect is difficult[21,22]. Methods for measuring performance in higher education have less variability, yet there is no clear indication that offering mindfulness training to university students improves their academic performance[23].

The mechanisms underlying any effect of mindfulness on work performance are also yet to be determined while two mechanistic pathways stand out that could explain such an effect of MBPs. First, positive effects of MBPs on mental well-being are well-established[7–9], and mental well-being is linked to better work performance[24,25]. Conversely, mental health problems decrease employees' performance[26–28], particularly if these problems are poorly managed[29]. However, an indirect effect of MBPs on workplace performance via improved mental well-being has yet to be evaluated.

A second potential mechanism could be an improved cognitive control over mental activity, which allows one to prioritise current task-relevant goals[30,31]. There are three potential

facets of cognitive control that may be improved by MBPs: (a) shifting, that is, the ability to switch between multiple tasks; (b) updating, or the ability to frequently refresh information in working memory to ensure a currently relevant record of information; and (c) inhibition: deliberately hindering dominant or automatic responses that are irrelevant to the task at hand[32]. Improved cognitive control, in turn, may lead to better performance on workplace tasks[11,33].

Mindfulness has been shown to have a small effect on cognitive control[34,35]. A recent meta-analysis analysing outcomes of randomised controlled trials (RCTs) measuring the effects of cognitive control in MBPs for healthy participants found a small overall effect of Hedge's $g$=0.2[35]. However, we know little about how these changes in cognitive control manifest in the workplace[21]. While mindfulness may improve performance on tasks closely related to the practice, such as counting breaths[36], it may not extend to other tasks, such as those completed at work[37].

Furthermore, to date, research has primarily focussed on the impact of mindfulness on cognitive control over emotionally neutral information. Yet, much of the everyday mental activity that we seek to regulate, is emotionally positive or negative[38,39]. In the two meta-analysis of MBPs' effects on cognitive control published to date[34,35], only one identified study used emotional stimuli within an cognitive control task. This study reported a null-effect of meditation on cognitive control measured via an attention network test when comparing negative and neutral conditions[40]. It is important to note that this study[40] was likely underpowered.

At work, it is arguably beneficial to inhibit emotional thoughts (e.g., worrying about a recent argument with your spouse) that are irrelevant to the task at hand (e.g., writing a report). A reduced ability to inhibit internal emotional stimuli may interfere with our ability to maintain focus on workplace tasks. There is evidence that emotional stimuli inhibit cognitive control, when measured using the Stop-Signal Task[41–43]. As mindful meditation trains the ability to move away from thoughts and images of negative emotional valence, practicing mindfulness may enhance cognitive control over emotional mental events[44]. It is therefore important to determine whether MBPs improve workplace performance via enhancement of cognitive control skills such as the ability to move away from negative stimuli (e.g., worries about task performance) or to decentre from negative mental content[45,46] and refocus attention on the task at hand[22].

Understanding the mechanisms underlying effects of MBPs on work performance would (a) help to design more targeted interventions, (b) improve our attempts to assess MBPs, by designing and selecting more stringent outcome measures and control interventions and (c) inform an understanding of for whom MBPs may be most effective, and in which context[47].

# Objectives

For peer review only

Current literature suggests that MBPs could improve work performance through increased mental well-being and/or cognitive control over emotional material. A definitive randomised controlled trial is needed to evaluate these potential mechanisms. However, methodological uncertainties and questions of acceptability and feasibility need clarification to inform the design of such a trial[48–50]. We aim to conduct a feasibility trial to clarify these uncertainties and complete a preliminary investigation of the relationships between mindfulness training, workplace performance and the proposed mechanisms of action.

This feasibility trial will:

1. Estimate the between-groups effect size for the effect of mindfulness, relative to a light exercise control condition, on our primary outcome of work performance, in order to inform power calculation for a larger trial;
2. Explore whether improved cognitive control and/or enhanced mental health could be potential mechanisms underlying the effect of mindfulness on work performance;
3. Assess the acceptability of the interventions and the study design by monitoring recruitment, retention, and adherence to the course;
4. Determine procedural feasibility of a later stage trial by evaluating the willingness of the participants to be randomised and other practical implications of running a randomised controlled trial at a workplace.

# METHODS

This protocol follows the guidelines for RCTs set by the SPIRIT 2013 statement[51] (SPIRIT checklist in Supplementary Materials). The study's prospective registration number at clinicaltrials.gov is NCT04631302.

# Study design

We will conduct a participant-level RCT. Employees will be randomly allocated in a 1:1 ratio to either of two parallel groups.

# Eligibility criteria

Eligibility to participate in this study will be self-reported. The employers who have agreed to participate in the study trade either in the publishing, electronics, or construction industry and employ thousands of employees in a variety of roles, mostly in desk-based occupations. Individuals can participate if they work for the employers taking part in this trial, are based in the UK, and are not currently on a long-term leave. We will recommend that a participant chooses not to join the study if they:

- are currently suffering from severe periods of anxiety, depression or hypomania/mania;
- are experiencing other severe mental illnesses;
- have had a recent bereavement or major loss;

For peer review only

- have already completed a mindfulness course or have meditated more than 10 hours in the past 10 years.

## Intervention condition: *Be Mindful* mindfulness-based programme

Participants in the Mindfulness condition will complete the *Be Mindful* pre-recorded online course by Wellmind Media. It incorporates elements from Mindfulness-Based Stress Reduction[52] and Mindfulness-Based Cognitive Therapy[53]. Course materials and instructional videos are accessed through a website (http://www.bemindfulonline.com).

The four-week course consists of 10 sessions led by two teachers, one female, one male. Participants are taught to use formal meditations (focusing attention on the practice of meditation) as well as informal mindfulness techniques, such as mindful walking and mindful eating. Daily homework includes a formal mediation practice with the assistance of video/audio recordings (up to 30 minutes), and one or two informal exercises per day (see Table 1 for an overview). Every week, participants receive e-mails motivating them to practice and informing them when the next module is available. As this is a feasibility examination for a pragmatic trial, no modifications to the procedures to maintain adherence to the intervention will be implemented.

## Control condition: Light physical exercise

The four-week control condition involves light exercises aimed at increasing mobility, reducing stiffness, improving blood circulation, and avoiding pain or repetitive strain injuries that may result from sedentary or repetitive tasks common in office environments. The pre-recorded exercises will include whole-body slightly aerobic exercises such as rotation of joints and stretching. The course was developed by JG, a public health doctor, together with an expert in body posture.

The control condition course is designed to match the intervention condition in overall time commitment, and the frequency of interaction with the participant (see Table 1 for an overview). It replicates the encouraged use of short breaks throughout the workday to focus on well-being, as in the intervention condition. While both the MBP and control interventions have been shown to reduce stress, depression and anxiety[54–56], only mindfulness is expected to improve cognitive control skills. This control condition will therefore help to distinguish between the different pathways through which work performance may improve.

Table 1. Comparison of the intervention and control group

| Condition | Intervention: Be Mindful | Control: Light physical exercise |
|---|---|---|

For peer review only

| | | |
|---|---|---|
| **Number of sessions in total** | 10 | 28 |
| **Online coursework frequency** | Twice weekly | Daily |
| **Typical session and its length** | Self-paced. Includes videos (average of 3-4 minutes in total per session), self-reflection exercises and brief reading tasks. | Videos of 10-13 minutes. |
| **Homework frequency** | Daily | Daily |
| **Typical assignment** | A formal meditation (10-30 minutes) and shorter task such as journaling or noticing. The frequency of the latter varies from daily to once a week. | Using the exercises while taking brief breaks during the day. |
| **Reminders to encourage practicing** | 4 times a week | 4 times a week |

# Data collection

Data collection will take place at baseline (T0), after the courses finish (T1) and 12 weeks after completing the courses (T3) (see **Error! Reference source not found.**). Additionally, a brief questionnaire will be sent to the participants each workday. Data collected at T1 will be considered as the primary end-point of interest.

Figure 1 should be inserted here

# Outcomes

## Feasibility, acceptability, and procedural outcomes

To determine feasibility of a later-stage trial, we will examine descriptive statistics to:

1. Estimate between-condition effect sizes:
    a. for the primary outcome, to inform a power calculation for a later-stage trial;
    b. for the cognitive control outcomes, to determine suitability of these measures to index mechanisms of interest.
2. Determine feasibility of running a later-stage trial by monitoring recruitment and retention, including timing of measurements (by indexing percentage of participants completing each time point), and potential contamination issues, most notably, measuring the extent to which participants talked about their course with participants from the other arm;
3. Acceptability of interventions by indexing which course the participants would have preferred to be randomised to, and their regularity in engaging in exercise and mindfulness;

For peer review only

4. Procedural uncertainties, for example, by exploring the suitability of our primary measure in indexing our primary outcome. To this end, we have introduced several work-related outcomes (see Secondary Outcomes);
5. Potential covariates influencing key outcomes which may need to be considered in design of the later-stage trial, including:
    a. participant mental and physical health at baseline;
    b. importance of job to participants' identity at baseline.

## Primary outcome: Work performance

Work performance will be measured by using the 25-item Work Role Functioning Questionnaire's[57] updated version[58], to capture perceived difficulties in meeting work demands. Items are rated on a 5-point scale ('difficult all the time' to 'difficult none of the time'), with higher scores indicating better functioning. A 6th option denotes 'does not apply to my job'. The questionnaire has not been validated in English. Validations completed in Dutch[58], Spanish[59], and Norwegian[60] have shown good internal consistency (Cronbach alphas 0.7-0.9)[58–60], and test-retest reliability (ICC=0.66, 95% CI: 0.54-0.76 for the total score)[58]. The WRFQ features four subscales: work scheduling and output demands ($\alpha$=0.92), physical demands ($\alpha$=0.92), mental and social demands ($\alpha$=0.91), and flexibility demands ($\alpha$=0.96)[58]. The WRFQ has been shown to possess decent convergent validity, correlating with similar measures including the Utrecht Work Engagement Scale[61] (r=0.304)[58], Work Ability Index[62] (r=0.468)[58]. The primary endpoint in this trial will be the post-intervention measurement. Feasibility of using the 12-week follow-up as the main outcome end-point in the later-stage trial, will be assessed.

## Secondary outcomes

### *Work-related outcomes*

Those who self-report experiencing mental or physical health problems will be asked to fill in the Work and Social Adjustment Scale[63]. The scale is widely used in the NHS psychology services in England and has good internal consistency, $\alpha$=0.82[64].

To get a better understanding of daily fluctuations that may occur in work engagement, participants will be asked to complete a 5-item version of the Work Role Functioning Questionnaire[65] each workday afternoon. Items are rated the same as in the full Work Role Functioning Questionnaire.

### *Cognitive control mechanisms*

Two online computerised cognitive tasks will be used, to index our potential executive function mechanisms of interest. Affective cognitive control will be assessed using the Emotional Stop-Signal Task[66]. At the beginning of each trial within the task, a negative or a neutral image appears, followed by a go-signal (left or right arrow). Participants need to respond with a corresponding key-press. On a minority of trials (20%), the go-signal is

For peer review only

followed by a stop-signal (upwards arrow) in which a go-response is required to be inhibited. Reaction times (in both, go- and stop-trials), response accuracy (failure or success in inhibiting response) and variability in reaction time throughout the task (a proxy for the ability to overcome errors) will be measured. The main outcome of interest is the response time in stop-trials.

Participant's ability to track dynamic changes in their environment and alter their response strategies will be measured using an affective modification of the probabilistic reversal learning task[68]. The task will consist of 6 phases, 3 for the neutral and 3 for the negative condition. Each trial will begin with a negative or a neural image from the International Affective Picture System (IAPS)[67]. Next, pairs of stimuli (A-B or C-D) will be presented. Participants must select a stimulus with a key-press. In each pair, one of the stimuli is more likely to be rewarded (e.g., selecting A or C is reinforced on 80% of trials). Feedback is presented after each response. Through trial-and-error, participants learn which stimuli are more frequently rewarded. After a certain number of trials (a phase), the contingency of reinforcement switches. In Phase 2, the other stimulus in the pair is more frequently reinforced (e.g., instead of A, B is now reinforced on 80% of trials). In Phase 3, the reinforcement is switched again. Reaction times and response accuracy (i.e., selecting the reinforced stimulus) will be recorded. The main outcome of interest will be changes in learning performance indexed via the proportion of correct responses.

## *Other outcomes of interest*

### Well-being

Subjective mental well-being will be measured with the Short Warwick-Edinburgh Mental Well-being Scale (SWEMWBS), a 7-item questionnaire designed to capture a broad concept of well-being[69]. In SWEMWBS, items are scored on a scale of 1-5 ('none of the time'…'all of the time'), with higher scores suggesting better mental well-being. The SWEMWBS internal consistency was α=0.84 in a study in the UK general population (n=27,169)[70].

### Stress

The Perceived Stress Scale (PSS) measures the extent to which the individual has perceived events as uncontrollable and overwhelming. The PSS consists of 10 items, answered on a scale of 0-4, higher scores indicate higher stress levels. The PSS possesses good internal consistency, α=0.84-0.86[71].

### Depression

The Patient Health Questionnaire (PHQ-9)[72] is used to assess depression. It consists of 9 items answered using a scale from 0-3, and a further item asking about the level of difficulty associated with any checked off items. Total scores range from 0-27 with cut-off points for depression at 5, 10, 15 and 20 for mild, moderate, moderately severe and severe depression,

For peer review only

respectively[72]. A PHQ-9 score of at least 10 has been found to have 88% sensitivity and 88% specificity for major depression[72].

### Anxiety

The General Anxiety Disorder 7-item Scale (GAD-7)[73] assesses anxiety and has good reliability and validity[74]. The items are answered using a scale from 0-3, yielding total scores between 0 and 21 with cut-offs at 5, 10 and 15 for mild, moderate and severe anxiety, respectively[73]. The scale's internal consistency is α=.92. A total score of 10 has a 89% sensitivity and 82% specificity for generalised anxiety disorder[73].

### *Mindfulness-related outcomes*

The following will be administered to ensure that the MBP does increase mindfulness more than the control condition.

### Decentering

The Experiences Questionnaire (EQ)[45] is an 11-item measure of decentering. The items were generated to represent the changes believed to occur due to mindfulness practice, including the extent to which one's self-identity depends on one's thoughts, nonreactivity to negative experiences, and self-compassion. Statements are rated on a 5-point scale ('never' to 'all the time'), with higher scores reflecting higher levels of decentering. The scale's internal consistency is α=.81–.84[45].

### Mindfulness

Mindful Attention Awareness Scale (MAAS)[75] is a self-report questionnaire consisting of 15 items designed to assess a core characteristic of mindfulness – a receptive state of mind in which attention simply observes what is taking place. Items are rated using a 6-point Likert scale ('almost always' to 'almost never'), with higher scores indicating more mindfulness. The internal consistency of MAAS is α=.87[75].

For peer review only

## Sample size

One of the procedural uncertainties limiting the design of a fully-powered trial is the size of the effect on the main outcome in interest. As a traditional power calculation is unfeasible given the lack of previous data, we seek to determine the likely effect size in this study, to inform a later-phase trial.

We aim to recruit 240 participants. A fully online design may cause a high loss to follow-up; a systematic review of internet-based RCTs found the average attrition rate to be 47% at post-intervention[76]. Based on this, we have selected a sample size which we anticipate will yield complete data for 128 participants at our primary end-point of post-intervention (64 per arm) and 68 participants at follow-up (34 per arm). In clinical research with lower attrition

rates, feasibility trials tend to recruit 36 participants[77]. Considering high risk of attrition and the considerable uncertainties regarding the feasibility of the trial, we estimate that our sample size is optimal to examine the feasibility of procedures and provide a reliable estimate of effect size.

# Study procedures

## Recruitment

Employers who have agreed to collaborate in the research project, have taken an active role in shaping the recruitment process to their organisational customs. The invitation, sent via web-based communication services used by the employer, will have a link to the participant information sheet and consent form. Initial consent taking started in November 2020. Participants, irrespective of the time they consent, received access to baseline measures from the 23rd February 2021.

## Inducements for participation

There will be no inducements for completing either of the interventions. As a token of appreciation for completing the study assessments, participants will be given £10 at post-intervention and £15 at follow-up time points in the form of retail vouchers.

## Randomisation procedure

After the participants have completed all baseline measurements, they will be randomised to either the mindfulness or the light physical exercise arm, stratified by employer. The randomisation process will be automated using REDCap, a platform for questionnaire data collection[78,79]. The allocation tables were generated with randomizeR package[80] in R using randomised permuted block randomisation with pre-specified seeds for reproducibility. The code is available at GitHub[81].

Participants will not be blind to their allocation. The primary analysis will be completed by a statistician (PW) blind to intervention allocation.

# Public involvement

The study's design has been formed by feedback from the employers participating in the study, including the perceived utility of the interventions, recruitment procedure and its timing, study materials, incentives for participation and outcomes. Changes to the initial design were proposed, some of which were implemented (e.g., offering vouchers rather than cash; channels and timing for recruitment).

# Statistical methods

For peer review only

Central tendencies, dispersion, and data missingness will be reported for all time points. At baseline, descriptive statistics will be presented overall and by group allocation. At following timepoints, outcomes will be reported by group.

Any significance testing, though not the focus of this trial, will follow the intention-to-treat principle. For the primary outcome, a linear multiple regression model will compare the WRFQ total score between trial arms at post-intervention, adjusted for baseline WRFQ and employer. Multiple imputation will be used to account for missing data. Further exploratory analysis will employ the same approach for other outcome measures at post-intervention and 12-week follow-up. Mediation analysis techniques will be employed to assess the suggested mechanistic pathways. Effect sizes obtained in these analyses will be used to inform a potential later-stage trial and are the focus of this trial, rather than statistical significance.

For the secondary outcomes, mixed model repeated measures analysis will be performed using the daily monitoring of work performance to study changes between arms during the intervention. The analysis will also compare different work performance measures. Again, the focus of this trial is on obtaining an estimate of likely effect sizes, rather than statistical significance.

## Data monitoring and adverse events

An Independent Study Steering group has been established to monitor data and advise the conduct of the study to ensure participant safety and integrity of research. We have established the following safeguards[83,84]:

1. Participants are made aware they may request a consultation with a clinical psychologist if they feel uncomfortable with the study or experience discomfort they associate with the interventions.

2. Where participants' responses to PHQ9 (depression) or GAD7 (anxiety) are above clinical cut-off scores (≥20 and ≥15, respectively), a warning is automatically sent to the researcher. For PHQ9, the alert is also triggered when the participant score is >0 on the self-harm item. The researcher (MV) will then consult the clinical psychologist who will contact the participant.

3. Participants wishing to leave the study will be encouraged to let the study team know why they have chosen to do so.

Any adverse events discovered through the mechanisms listed above will be discussed with the Independent Study Steering group who may decide whether they are attributable to the interventions (i.e., adverse effects) and any subsequent course of action.

# ETHICS AND DISSEMINATION

For peer review only - http://bmjopen.bmj.com/site/about/guidelines.xhtml

The trial has received approval from the Cambridge Psychology Research Ethics Committee PRE.2020.072.

## Consent

The consent form states eligibility criteria and the circumstances in which we recommend not to participate in the study. Participants are invited to join virtual information sessions or e-mail the study team should they have any questions.

Information about accessing mental health support services is made available to anyone visiting the participation information website and e-mailed to those who consent to the study. Only those who consent to participate will receive the link to baseline measurements.

## Data management

Data will be collected and curated using the Research Electronic Data Capture (REDCap)[78,79], the Cohort Management System (CMS) and JATOS[85]. Anonymised data will be shared for research purposes upon request, in line with open science principles. All personally identifiable data will be separated from study data and stored on separate encrypted servers.

## Dissemination policy

Findings will be submitted to peer-review journals. Authorship in publications will be based on the International Committee of Medical Journal Editors' criteria. We will also send a lay summary of the results to the participating employers and participants.

# ACKNOWLEDGEMENTS

The study team would like to thank Becky Gilbert, Camilla Nord, Marc Bennett, Rachel Knight, and Tierney Lee for their role in designing the cognitive tasks and Markus Hausammann for substantially contributing to the design of the study logo.

# CONTRIBUTORS

MV, CH, JG and TD developed the idea for the trial and applied for funding. MV, CH and JG drafted the protocol which was then revised through discussions with TD, the Emotion study group at MRC CBU and the participating employers. The analysis plan was devised by MV, CH, JG and PW. MV is the lead researcher. CH and JG are supervising the research. The trial is sponsored by University of Cambridge.

For peer review only

FUNDING STATEMENT

This study is supported by MRC Cognition and Brain Sciences Unit, MV is supported by Kristjan Jaak degree studies abroad scholarship by Estonia's Education and Youth Board, JG by the National Institute for Health Research (NIHR) Applied Research Collaboration East of England, CH by the Economic and Social Research Council and TD by the UK Medical Research Council (Grant reference: SUAG/043 G101400) and Wellcome (Grant reference: 104908/Z/14/Z, 107496/Z/15/Z). The views expressed are those of the authors and not necessarily those of the National Health Service, the NIHR or the Department of Health and Social Care.

# COMPETING INTERESTS' STATEMENT

None declared.

For peer review only

# REFERENCES


1 James SL, Abate D, Abate KH, *et al.* Global, regional, and national incidence, prevalence, and years lived with disability for 354 diseases and injuries for 195 countries and territories, 1990–2017: a systematic analysis for the Global Burden of Disease Study 2017. *The Lancet* 2018;**392**:1789–858. doi:10.1016/S0140-6736(18)32279-7

2 OECD.Stat. Employment: Time spent in paid and unpaid work, by sex. https://stats.oecd.org/index.aspx?queryid=54757

3 Office for National Statistics. Awake time per day in the United Kingdom (UK) in 2014, by age and gender. Statista. 2014.https://www.statista.com/statistics/326907/awake-time-per-day-uk-by-age-and-gender/ (accessed 15 Jul 2020).

4 Health and Safety Executive. Work-related stress, anxiety or depression statistics in Great Britain, 2019. Health and Safety Executive 2019. https://www.hse.gov.uk/statistics/causdis/stress.pdf (accessed 12 May 2020).

5 Deloitte UK. Mental Health and Employers: Refreshing the case for investment. Deloitte 2020. https://www2.deloitte.com/content/dam/Deloitte/uk/Documents/consultancy/deloitte-uk-mental-health-and-employers.pdf

6 Kabat-Zinn J. Mindfulness-based interventions in context: Past, present, and future. *Clin Psychol Sci Pract* 2003;**10**:144–56. doi:10.1093/clipsy/bpg016

7 Khoury B, Sharma M, Rush SE, *et al.* Mindfulness-based stress reduction for healthy individuals: A meta-analysis. *J Psychosom Res* 2015;**78**:519–28. doi:10.1016/j.jpsychores.2015.03.009

8 Galante J, Friedrich C, Dawson AF, *et al.* Mindfulness-based programmes for mental health promotion in adults in nonclinical settings: A systematic review and meta-analysis of randomised controlled trials. *PLOS Med* 2021;**18**:e1003481. doi:10.1371/journal.pmed.1003481

9 Spijkerman MPJ, Pots WTM, Bohlmeijer ET. Effectiveness of online mindfulness-based interventions in improving mental health: A review and meta-analysis of randomised controlled trials. *Clin Psychol Rev* 2016;**45**:102–14. doi:10.1016/j.cpr.2016.03.009

10 Shapiro SL, Astin JA, Bishop SR, *et al.* Mindfulness-Based Stress Reduction for Health Care Professionals: Results From a Randomized Trial. *Int J Stress Manag* 2005;**12**:164–76. doi:10.1037/1072-5245.12.2.164

11 Dane E. Paying Attention to Mindfulness and Its Effects on Task Performance in the Workplace. *J Manag* 2011;**37**:997–1018. doi:10.1177/0149206310367948

12 Shapiro SL, Wang MC, Peltason EH. What is mindfulness, and why should organizations care about it? In: Reb J, Atkins PWB, eds. *Mindfulness in Organizations*. Cambridge: : Cambridge University Press 2015. 17–41. doi:10.1017/CBO9781107587793.004


For peer review only

13 Aikens KA, Astin J, Pelletier KR, *et al.* Mindfulness Goes to Work: Impact of an Online Workplace Intervention. *J Occup Environ Med* 2014;**56**:721–31. doi:10.1097/JOM.0000000000000209

14 Wilson DM. *Effects of mindfulness-based art processing (MBAP) on the well-being and job performance of working adults: Evaluating a novel intervention*. 2012.

15 Klatt M, Steinberg B, Duchemin A-M. Mindfulness in Motion (MIM): An Onsite Mindfulness Based Intervention (MBI) for Chronically High Stress Work Environments to Increase Resiliency and Work Engagement. *JoVE J Vis Exp* 2015;:e52359. doi:10.3791/52359

16 van Berkel J, Boot CRL, Proper KI, *et al.* Effectiveness of a Worksite Mindfulness-Related Multi-Component Health Promotion Intervention on Work Engagement and Mental Health: Results of a Randomized Controlled Trial. *PLoS ONE* 2014;**9**:e84118. doi:10.1371/journal.pone.0084118

17 van Dongen JM, van Berkel J, Boot CRL, *et al.* Long-Term Cost-Effectiveness and Return-on-Investment of a Mindfulness-Based Worksite Intervention: Results of a Randomized Controlled Trial. *J Occup Environ Med* 2016;**58**:550–60. doi:10.1097/JOM.0000000000000736

18 Bartlett L, Lovell P, Otahal P, *et al.* Acceptability, Feasibility, and Efficacy of a Workplace Mindfulness Program for Public Sector Employees: a Pilot Randomized Controlled Trial with Informant Reports. *Mindfulness* 2017;**8**:639–54. doi:10.1007/s12671-016-0643-4

19 Verweij H, van Ravesteijn H, van Hooff MLM, *et al.* Mindfulness-Based Stress Reduction for Residents: A Randomized Controlled Trial. *J Gen Intern Med* 2018;**33**:429–36. doi:10.1007/s11606-017-4249-x

20 Luken M, Sammons A. Systematic Review of Mindfulness Practice for Reducing Job Burnout. *Am J Occup Ther* 2016;**70**:7002250020p1–10. doi:10.5014/ajot.2016.016956

21 Bartlett L, Martin A, Neil AL, *et al.* A systematic review and meta-analysis of workplace mindfulness training randomized controlled trials. *J Occup Health Psychol* 2019;**24**:108–26. doi:10.1037/ocp0000146

22 Jamieson SD, Tuckey MR. Mindfulness interventions in the workplace: A critique of the current state of the literature. *J Occup Health Psychol* 2017;**22**:180–93. doi:10.1037/ocp0000048

23 Dawson AF, Brown WW, Anderson J, *et al.* Mindfulness-Based Interventions for University Students: A Systematic Review and Meta-Analysis of Randomised Controlled Trials. *Appl Psychol Health Well-Being* 2019;**12**.https://onlinelibrary.wiley.com/doi/abs/10.1111/aphw.12188 (accessed 24 Feb 2020).

24 García-Buades ME, Peiró JM, Montañez-Juan MI, *et al.* Happy-Productive Teams and Work Units: A Systematic Review of the 'Happy-Productive Worker Thesis.' *Int J Environ Res Public Health* 2020;**17**. doi:10.3390/ijerph17010069

For peer review only

25 Montano D, Reeske A, Franke F, *et al.* Leadership, followers' mental health and job performance in organizations: A comprehensive meta-analysis from an occupational health perspective. *J Organ Behav* 2017;**38**:327–50. doi:10.1002/job.2124

26 Alonso J, Petukhova M, Vilagut G, *et al.* Days out of role due to common physical and mental conditions: results from the WHO World Mental Health surveys. *Mol Psychiatry* Published Online First: 2011. doi:10.1038/mp.2010.101

27 Collins JJ, Baase CM, Sharda CE, *et al.* The Assessment of Chronic Health Conditions on Work Performance, Absence, and Total Economic Impact for Employers. *J Occup Environ Med* 2005;**47**:547–57. doi:10.1097/01.jom.0000166864.58664.29

28 Stewart WF, Ricci JA, Chee E, *et al.* Cost of Lost Productive Work Time Among US Workers With Depression. *JAMA* 2003;**289**:3135–44. doi:10.1001/jama.289.23.3135

29 Rost K, Smith JL, Dickinson M. The Effect of Improving Primary Care Depression Management on Employee Absenteeism and Productivity A Randomized Trial. *Med Care* 2004;**42**:1202–10.

30 Schweizer S, Satpute AB, Atzil S, *et al.* The impact of affective information on working memory: A pair of meta-analytic reviews of behavioral and neuroimaging evidence. *Psychol Bull* 2019;**145**:566–609. doi:10.1037/bul0000193

31 Ionescu T. Exploring the nature of cognitive flexibility. *New Ideas Psychol* 2012;**30**:190–200. doi:10.1016/j.newideapsych.2011.11.001

32 Miyake A, Friedman NP, Emerson MJ, *et al.* The Unity and Diversity of Executive Functions and Their Contributions to Complex "Frontal Lobe" Tasks: A Latent Variable Analysis. *Cognit Psychol* 2000;**41**:49–100. doi:10.1006/cogp.1999.0734

33 Chaskalson M. *The mindful workplace: developing resilient individuals and resonant organizations with MBSR*. Chichester, West Sussex ; Malden, MA: : Wiley-Blackwell 2011.

34 Cásedas L, Pirruccio V, Vadillo MA, *et al.* Does Mindfulness Meditation Training Enhance Executive Control? A Systematic Review and Meta-Analysis of Randomized Controlled Trials in Adults. *Mindfulness* 2020;**11**:411–24. doi:10.1007/s12671-019-01279-4

35 Yakobi O, Smilek D, Danckert J. The Effects of Mindfulness Meditation on Attention, Executive Control and Working Memory in Healthy Adults: A Meta-analysis of Randomized Controlled Trials. *Cogn Ther Res* Published Online First: 4 January 2021. doi:10.1007/s10608-020-10177-2

36 Levinson DB, Stoll EL, Kindy SD, *et al.* A mind you can count on: Validating breath counting as a behavioral measure of mindfulness. *Front Psychol* 2014;**5**:1–10. doi:10.3389/fpsyg.2014.01202

37 Simons DJ, Boot WR, Charness N, *et al.* Do "brain-training" programs work? *Psychol Sci Public Interest Suppl* 2016;**17**:103–86. doi:10.1177/1529100616661983

For peer review only

38 Killingsworth MA, Gilbert DT. A Wandering Mind Is an Unhappy Mind. *Science* 2010;**330**:932–932. doi:10.1126/science.1192439

39 Kragel PA, Knodt AR, Hariri AR, *et al.* Decoding Spontaneous Emotional States in the Human Brain. *PLOS Biol* 2016;**14**:e2000106. doi:10.1371/journal.pbio.2000106

40 Ainsworth B, Eddershaw R, Meron D, *et al.* The effect of focused attention and open monitoring meditation on attention network function in healthy volunteers. *Psychiatry Res* 2013;**210**:1226–31. doi:10.1016/j.psychres.2013.09.002

41 Verbruggen F, De Houwer J. Do emotional stimuli interfere with response inhibition? Evidence from the stop signal paradigm. *Cogn Emot* 2007;**21**:391–403. doi:10.1080/02699930600625081

42 Kalanthroff E, Cohen N, Henik A. Stop feeling: inhibition of emotional interference following stop-signal trials. *Front Hum Neurosci* 2013;**7**. doi:10.3389/fnhum.2013.00078

43 Herbert C, Sütterlin S. Response Inhibition and Memory Retrieval of Emotional Target Words: Evidence from an Emotional Stop-Signal Task. *J Behav Brain Sci* 2011;**1**. doi:10.4236/jbbs.2011.13020

44 Crane RS, Brewer J, Feldman C, *et al.* What defines mindfulness-based programs? The warp and the weft. *Psychol Med* 2017;**47**:990–9. doi:10.1017/S0033291716003317

45 Fresco DM, Moore MT, van Dulmen MHM, *et al.* Initial Psychometric Properties of the Experiences Questionnaire: Validation of a Self-Report Measure of Decentering. *Behav Ther* 2007;**38**:234–46. doi:10.1016/j.beth.2006.08.003

46 Safran JD, Segal ZV. *Interpersonal process in cognitive therapy*. 1st softcover ed. Northvale, N.J: : Jason Aronson 1996.

47 Nielsen L, Riddle M, King JW, *et al.* The NIH Science of Behavior Change Program: Transforming the science through a focus on mechanisms of change. *Behav Res Ther* 2018;**101**:3–11. doi:10.1016/j.brat.2017.07.002

48 Bowen DJ, Kreuter M, Spring B, *et al.* How We Design Feasibility Studies. *Am J Prev Med* 2009;**36**:452–7. doi:10.1016/j.amepre.2009.02.002

49 Hallingberg B, Turley R, Segrott J, *et al.* Exploratory studies to decide whether and how to proceed with full-scale evaluations of public health interventions: a systematic review of guidance. *Pilot Feasibility Stud* 2018;**4**:104. doi:10.1186/s40814-018-0290-8

50 Medical Research Council. Developing and evaluating complex interventions. 2008. www.mrc.ac.uk/complexinterventionsguidance (accessed 17 Jun 2020).

51 Chan A-W, Tetzlaff JM, Altman DG, *et al.* SPIRIT 2013 Statement: Defining Standard Protocol Items for Clinical Trials. *Ann Intern Med* 2013;**158**:200–7. doi:10.7326/0003-4819-158-3-201302050-00583

For peer review only

52 Kabat-Zinn J. *Full catastrophe living: how to cope with stress, pain and illness using mindfulness meditation / by Jon Kabat-Zinn.* Revised an. London: Piatkus, c2013. 2013.

53 Segal ZV, Williams JMG, Teasdale JD. *Mindfulness-based cognitive therapy for depression*. 2nd ed. New York: : Guilford Press 2013.

54 Galante J, Bekkers M-J, Mitchell C, *et al.* Loving-Kindness Meditation Effects on Well-Being and Altruism: A Mixed-Methods Online RCT. *Appl Psychol Health Well-Being* 2016;**8**:322–50. doi:10.1111/aphw.12074

55 Querstret D, Cropley M, Fife-Schaw C. The Effects of an Online Mindfulness Intervention on Perceived Stress, Depression and Anxiety in a Non-clinical Sample: A Randomised Waitlist Control Trial. *Mindfulness* 2018;**9**:1825–36. doi:10.1007/s12671-018-0925-0

56 Krusche A, Cyhlarova E, Williams JMG. Mindfulness online: An evaluation of the feasibility of a web-based mindfulness course for stress, anxiety and depression. *BMJ Open* 2013;**3**:1–10. doi:10.1136/bmjopen-2013-003498

57 Amick BC, Lerner D, Rogers WH, *et al.* A Review of Health-Related Work Outcome Measures and Their Uses, and Recommended Measures: *Spine* 2000;**25**:3152–60. doi:10.1097/00007632-200012150-00010

58 Abma FI, van der Klink JJL, Bültmann U. The Work Role Functioning Questionnaire 2.0 (Dutch Version): Examination of its Reliability, Validity and Responsiveness in the General Working Population. *J Occup Rehabil* 2013;**23**:135–47. doi:10.1007/s10926-012-9379-8

59 Ramada JM, Serra C, Amick BC, *et al.* Reliability and Validity of the Work Role Functioning Questionnaire (Spanish Version). *J Occup Rehabil* 2014;**24**:640–9. doi:10.1007/s10926-013-9495-0

60 Johansen T, Lund T, Jensen C, *et al.* Cross-cultural adaptation of the Work Role Functioning Questionnaire 2.0 to Norwegian and Danish. *Work* 2018;**59**:471–8. doi:10.3233/WOR-182705

61 Schaufeli WB, Bakker AB. Utrecht Work Engagement Scale. Preliminary Manual. 2004.

62 Ilmarinen J. The Work Ability Index (WAI). *Occup Med* 2007;**57**:160–160. doi:10.1093/occmed/kqm008

63 Marks IM, Bird J. *Behavioural psychotherapy: Maudsley pocket book of clinical management*. 6th ed. Bristol: : Wright 1986.

64 Zahra D, Qureshi A, Henley W, *et al.* The work and social adjustment scale: Reliability, sensitivity and value. *Int J Psychiatry Clin Pract* 2014;**18**:131–8. doi:10.3109/13651501.2014.894072

65 Abma FI, Bjorner JB, Amick BC, *et al.* Two valid and reliable work role functioning questionnaire short versions were developed: WRFQ 5 and WRFQ 10. *J Clin Epidemiol* 2019;**105**:101–11. doi:10.1016/j.jclinepi.2018.09.005

For peer review only

66 Verbruggen F, Logan GD. Response inhibition in the stop-signal paradigm. *Trends Cogn Sci* 2008;**12**:418–24. doi:10.1016/j.tics.2008.07.005

67 Bradley MM, Lang PJ. International Affective Picture System. In: Zeigler-Hill V, Shackelford TK, eds. *Encyclopedia of Personality and Individual Differences*. Cham: : Springer International Publishing 2017. 1–4. doi:10.1007/978-3-319-28099-8_42-1

68 Cools R, Clark L, Owen AM, *et al.* Defining the Neural Mechanisms of Probabilistic Reversal Learning Using Event-Related Functional Magnetic Resonance Imaging. *J Neurosci* 2002;**22**:4563–7. doi:10.1523/JNEUROSCI.22-11-04563.2002

69 Tennant R, Hiller L, Fishwick R, *et al.* The Warwick-Edinburgh Mental Well-being Scale (WEMWBS): Development and UK validation. *Health Qual Life Outcomes* 2007;**5**:63. doi:10.1186/1477-7525-5-63

70 Ng Fat L, Scholes S, Boniface S, *et al.* Evaluating and establishing national norms for mental wellbeing using the short Warwick–Edinburgh Mental Well-being Scale (SWEMWBS): findings from the Health Survey for England. *Qual Life Res* 2017;**26**:1129–44. doi:10.1007/s11136-016-1454-8

71 Cohen S, Kamarck T, Mermelstein R. A Global Measure of Perceived Stress. *J Health Soc Behav* 1983;**24**:385. doi:10.2307/2136404

72 Kroenke K, Spitzer RL, Williams JBW. The PHQ-9. *J Gen Intern Med* 2001;**16**:606–13. doi:10.1046/j.1525-1497.2001.016009606.x

73 Spitzer RL, Kroenke K, Williams JBW, *et al.* A Brief Measure for Assessing Generalized Anxiety Disorder: The GAD-7. *Arch Intern Med* 2006;**166**:1092–7. doi:10.1001/archinte.166.10.1092

74 Löwe B, Decker O, Müller S, *et al.* Validation and standardization of the Generalized Anxiety Disorder Screener (GAD-7) in the general population. *Med Care* 2008;**46**:266–74. doi:10.1097/MLR.0b013e318160d093

75 Brown KW, Ryan RM. The Benefits of Being Present: Mindfulness and Its Role in Psychological Well-Being. *J Pers Soc Psychol* 2003;**84**:822–48. doi:10.1037/0022-3514.84.4.822

76 Mathieu E, McGeechan K, Barratt A, *et al.* Internet-based randomized controlled trials: a systematic review. *J Am Med Inform Assoc* 2013;**20**:568–76. doi:10.1136/amiajnl-2012-001175

77 Billingham SA, Whitehead AL, Julious SA. An audit of sample sizes for pilot and feasibility trials being undertaken in the United Kingdom registered in the United Kingdom Clinical Research Network database. *BMC Med Res Methodol* 2013;**13**:104. doi:10.1186/1471-2288-13-104

78 Harris PA, Taylor R, Thielke R, *et al.* Research electronic data capture (REDCap)--a metadata-driven methodology and workflow process for providing translational research informatics support. *J Biomed Inform* 2009;**42**:377–81. doi:10.1016/j.jbi.2008.08.010


For peer review only

79 Harris PA, Taylor R, Minor BL, *et al.* The REDCap consortium: Building an international community of software platform partners. *J Biomed Inform* 2019;**95**:103208. doi:10.1016/j.jbi.2019.103208

80 Uschner D, Schindler D, Hilgers R-D, *et al.* randomizeR: An R package for the assessment and implementation of randomization in clinical trials. *J Stat Softw* 2018;**85**:1–22. doi:10.18637/jss.v085.i08

81 Vainre M. *Randomisation for the SWELL Trial*. 2021. https://github.com/mvainre/SWELL (accessed 21 Jan 2021).

82 Thabane L, Mbuagbaw L, Zhang S, *et al.* A tutorial on sensitivity analyses in clinical trials: the what, why, when and how. *BMC Med Res Methodol* 2013;**13**:92. doi:10.1186/1471-2288-13-92

83 Baer R, Crane C, Miller E, *et al.* Doing no harm in mindfulness-based programs: Conceptual issues and empirical findings. *Clin Psychol Rev* 2019;**71**:101–14. doi:10.1016/j.cpr.2019.01.001

84 Van Dam NT, Galante J. Underestimating harm in mindfulness-based stress reduction. *Psychol Med* 2020;:1–3. doi:10.1017/S003329172000447X

85 Lange K, Kühn S, Filevich E. "Just Another Tool for Online Studies" (JATOS): An Easy Solution for Setup and Management of Web Servers Supporting Online Studies. *PLOS ONE* 2015;**10**:e0130834. doi:10.1371/journal.pone.0130834


For peer review only

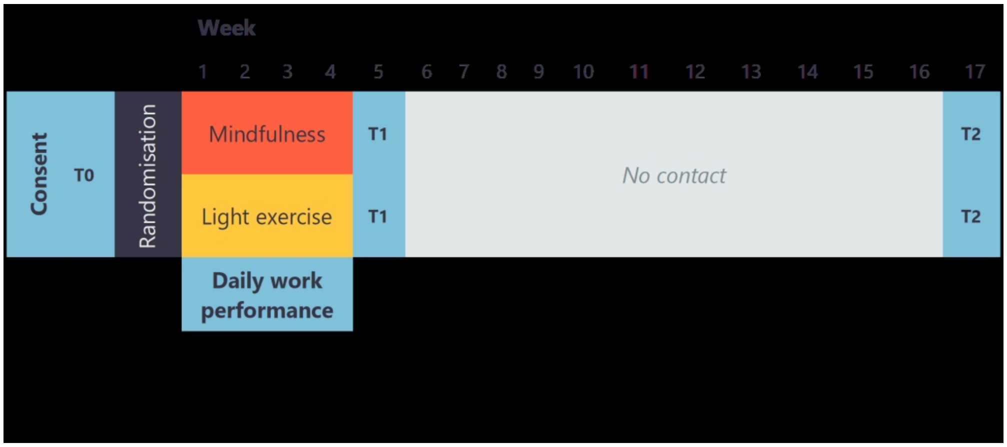


Figure 1. Study procedures and timeline

403x177mm (142 x 142 DPI)

STANDARD PROTOCOL ITEMS: RECOMMENDATIONS FOR INTERVENTIONAL TRIALS

SPIRIT 2013 Checklist: Recommended items to address in a clinical trial protocol and related documents*

| Section/item | ItemNo | Description | Where to find |
|---|---|---|---|
| **Administrative information** | | | |
| Title | 1 | Descriptive title identifying the study design, population, interventions, and, if applicable, trial acronym | Title page |
| Trial registration | 2a | Trial identifier and registry name. If not yet registered, name of intended registry | Abstract |
| | 2b | All items from the World Health Organization Trial Registration Data Set | Outlined in trial registration |
| Protocol version | 3 | Date and version identifier | Abstract |
| Funding | 4 | Sources and types of financial, material, and other support | Funding statement, p 15 |
| Roles and responsibilities | 5a | Names, affiliations, and roles of protocol contributors | Title page, Contributors, pp 15-16 |
| | 5b | Name and contact information for the trial sponsor | Contributors, trial registration |
| | 5c | Role of study sponsor and funders, if any, in study design; collection, management, analysis, and interpretation of data; writing of the report; and the decision to submit the report for publication, including whether they will have ultimate authority over any of these activities | Contributors, p 15 |



For peer review only - http://bmjopen.bmj.com/site/about/guidelines.xhtml

| | | | |
|---|---|---|---|
| | 5d | Composition, roles, and responsibilities of the coordinating centre, steering committee, endpoint adjudication committee, data management team, and other individuals or groups overseeing the trial, if applicable (see Item 21a for data monitoring committee) | pp 13-14 |
| **Introduction** | | | |
| Background and rationale | 6a | Description of research question and justification for undertaking the trial, including summary of relevant studies (published and unpublished) examining benefits and harms for each intervention | pp 5-6 |
| | 6b | Explanation for choice of comparators | p 7 |
| Objectives | 7 | Specific objectives or hypotheses | p 6 |
| Trial design | 8 | Description of trial design including type of trial (eg, parallel group, crossover, factorial, single group), allocation ratio, and framework (eg, superiority, equivalence, noninferiority, exploratory) | p 6 |
| **Methods: Participants, interventions, and outcomes** | | | |
| Study setting | 9 | Description of study settings (eg, community clinic, academic hospital) and list of countries where data will be collected. Reference to where list of study sites can be obtained | p 6 |
| Eligibility criteria | 10 | Inclusion and exclusion criteria for participants. If applicable, eligibility criteria for study centres and individuals who will perform the interventions (eg, surgeons, psychotherapists) | p 6 |
| Interventions | 11a | Interventions for each group with sufficient detail to allow replication, including how and when they will be administered | p 7 |
| | 11b | Criteria for discontinuing or modifying allocated interventions for a given trial participant (eg, drug dose change in response to harms, participant request, or improving/worsening disease) | p 7 and p 13 |



For peer review only - http://bmjopen.bmj.com/site/about/guidelines.xhtml

| | | | |
|---|---|---|---|
| | 11c | Strategies to improve adherence to intervention protocols, and any procedures for monitoring adherence (eg, drug tablet return, laboratory tests) | p 7 |
| | 11d | Relevant concomitant care and interventions that are permitted or prohibited during the trial | p 6 |
| Outcomes | 12 | Primary, secondary, and other outcomes, including the specific measurement variable (eg, systolic blood pressure), analysis metric (eg, change from baseline, final value, time to event), method of aggregation (eg, median, proportion), and time point for each outcome. Explanation of the clinical relevance of chosen efficacy and harm outcomes is strongly recommended | pp 8-11 |
| Participant timeline | 13 | Time schedule of enrolment, interventions (including any run-ins and washouts), assessments, and visits for participants. A schematic diagram is highly recommended (see Figure) | p 8 |
| Sample size | 14 | Estimated number of participants needed to achieve study objectives and how it was determined, including clinical and statistical assumptions supporting any sample size calculations | Sample size, p 11 |
| Recruitment | 15 | Strategies for achieving adequate participant enrolment to reach target sample size | Recruitment, p 12 |
| **Methods: Assignment of interventions (for controlled trials)** | | | |
| Allocation: | | | |
| Sequence generation | 16a | Method of generating the allocation sequence (eg, computer-generated random numbers), and list of any factors for stratification. To reduce predictability of a random sequence, details of any planned restriction (eg, blocking) should be provided in a separate document that is unavailable to those who enrol participants or assign interventions | Randomisation, p12 and GitHub |
| Allocation concealment mechanism | 16b | Mechanism of implementing the allocation sequence (eg, central telephone; sequentially numbered, opaque, sealed envelopes), describing any steps to conceal the sequence until interventions are assigned | Randomisation, p12 |

| | | | |
|---|---|---|---|
| Implementation | 16c | Who will generate the allocation sequence, who will enrol participants, and who will assign participants to interventions | Randomisation, p12 |
| Blinding (masking) | 17a | Who will be blinded after assignment to interventions (eg, trial participants, care providers, outcome assessors, data analysts), and how | Randomisation, p12 |
| | 17b | If blinded, circumstances under which unblinding is permissible, and procedure for revealing a participant's allocated intervention during the trial | Randomisation, p12 |
| **Methods: Data collection, management, and analysis** | | | |
| Data collection methods | 18a | Plans for assessment and collection of outcome, baseline, and other trial data, including any related processes to promote data quality (eg, duplicate measurements, training of assessors) and a description of study instruments (eg, questionnaires, laboratory tests) along with their reliability and validity, if known. Reference to where data collection forms can be found, if not in the protocol | p 8, Data management, p 14 |
| | 18b | Plans to promote participant retention and complete follow-up, including list of any outcome data to be collected for participants who discontinue or deviate from intervention protocols | p 12 |
| Data management | 19 | Plans for data entry, coding, security, and storage, including any related processes to promote data quality (eg, double data entry; range checks for data values). Reference to where details of data management procedures can be found, if not in the protocol | Data management, p 14 |
| Statistical methods | 20a | Statistical methods for analysing primary and secondary outcomes. Reference to where other details of the statistical analysis plan can be found, if not in the protocol | Statistical methods, p 13 |
| | 20b | Methods for any additional analyses (eg, subgroup and adjusted analyses) | Statistical methods, p 13 |
| | 20c | Definition of analysis population relating to protocol non-adherence (eg, as randomised analysis), and any statistical methods to handle missing data (eg, multiple imputation) | Statistical methods, p 13 |



For peer review only - http://bmjopen.bmj.com/site/about/guidelines.xhtml

**Methods: Monitoring**

| | | | |
|---|---|---|---|
| Data monitoring | 21a | Composition of data monitoring committee (DMC); summary of its role and reporting structure; statement of whether it is independent from the sponsor and competing interests; and reference to where further details about its charter can be found, if not in the protocol. Alternatively, an explanation of why a DMC is not needed | p 13 |
| | 21b | Description of any interim analyses and stopping guidelines, including who will have access to these interim results and make the final decision to terminate the trial | p 13-14 |
| Harms | 22 | Plans for collecting, assessing, reporting, and managing solicited and spontaneously reported adverse events and other unintended effects of trial interventions or trial conduct | p 13-14 |
| Auditing | 23 | Frequency and procedures for auditing trial conduct, if any, and whether the process will be independent from investigators and the sponsor | p 13-14 |

**Ethics and dissemination**

| | | | |
|---|---|---|---|
| Research ethics approval | 24 | Plans for seeking research ethics committee/institutional review board (REC/IRB) approval | p 14 |
| Protocol amendments | 25 | Plans for communicating important protocol modifications (eg, changes to eligibility criteria, outcomes, analyses) to relevant parties (eg, investigators, REC/IRBs, trial participants, trial registries, journals, regulators) | p 14 |
| Consent or assent | 26a | Who will obtain informed consent or assent from potential trial participants or authorised surrogates, and how (see Item 32) | p 14 |
| | 26b | Additional consent provisions for collection and use of participant data and biological specimens in ancillary studies, if applicable | NA |
| Confidentiality | 27 | How personal information about potential and enrolled participants will be collected, shared, and maintained in order to protect confidentiality before, during, and after the trial | p 14 |

review only



For peer review only - http://bmjopen.bmj.com/site/about/guidelines.xhtml

| | | | |
|---|---|---|---|
| Declaration of interests | 28 | Financial and other competing interests for principal investigators for the overall trial and each study site | p 15 |
| Access to data | 29 | Statement of who will have access to the final trial dataset, and disclosure of contractual agreements that limit such access for investigators | p 14 |
| Ancillary and post-trial care | 30 | Provisions, if any, for ancillary and post-trial care, and for compensation to those who suffer harm from trial participation | p 13-14 |
| Dissemination policy | 31a | Plans for investigators and sponsor to communicate trial results to participants, healthcare professionals, the public, and other relevant groups (eg, via publication, reporting in results databases, or other data sharing arrangements), including any publication restrictions | p 14 |
| | 31b | Authorship eligibility guidelines and any intended use of professional writers | p 14 |
| | 31c | Plans, if any, for granting public access to the full protocol, participant-level dataset, and statistical code | p 14 |
| **Appendices** | | | |
| Informed consent materials | 32 | Model consent form and other related documentation given to participants and authorised surrogates | See supplementary file 2 |
| Biological specimens | 33 | Plans for collection, laboratory evaluation, and storage of biological specimens for genetic or molecular analysis in the current trial and for future use in ancillary studies, if applicable | NA |

*It is strongly recommended that this checklist be read in conjunction with the SPIRIT 2013 Explanation & Elaboration for important clarification on the items. Amendments to the protocol should be tracked and dated. The SPIRIT checklist is copyrighted by the SPIRIT Group under the Creative Commons "Attribution-NonCommercial-NoDerivs 3.0 Unported" license.



For peer review only - http://bmjopen.bmj.com/site/about/guidelines.xhtml

# SUPPLEMENTARY MATERIAL 2: CONSENT FORM

**Thank you for considering taking part in this study. Our research depends entirely on the goodwill of potential volunteers such as you.**

**Where are you based?**
* must provide value

- ◯ In the UK
- ◯ Elsewhere

reset

**What's your work e-mail address?**
* must provide value

You'll be able to change the e-mail we use to contact you when the study starts.

**Please select the following boxes to indicate that you understand what participating in this study means.**

* must provide value — ◯ I confirm that I have read and understood the volunteer information above. reset

* must provide value — ◯ I understand I can ask questions by contacting the Principle Investigator, Maris Vainre, via e-mail. reset

* must provide value — ◯ I understand that my participation is voluntary. I am free to withdraw at any time without giving a reason, without penalty or affecting my rights. reset

* must provide value — ◯ I understand that the research data may be accessed by researchers working at or collaborating with the MRC Cognition and Brain Sciences Unit in similar ethically approved studies. At all times, my personal data will be kept confidential in accordance with data protection guidelines. reset

* must provide value — ◯ I understand that the research data may be accessed by researchers working at or collaborating with the MRC Cognition and Brain Sciences Unit in similar ethically approved studies. At all times, my personal data will be kept confidential in accordance with data protection guidelines. reset

* must provide value — ◯ I work at [Employer name] and am based in the UK reset

* must provide value — ◯ I am able to contribute between 3 to 4 hours a week over 4 weeks in January and February reset

* must provide value — ◯ I understand that I will be contacted again 3 months after the course finishes to follow-up on how things have been going for me reset

**If you're currently on parental or other long-term leave, we are not able to offer you a place in this study, sorry.**

**We recommend not to take part in this study if:**

- You are currently suffering from severe periods of anxiety, depression or hypomania;
- You are experiencing other severe mental illnesses;
- You have had a recent bereavement or major loss;
- You have already completed a mindfulness course or have meditated more than 10 hours in the past 10 years.

**Do you agree to take part in the study?**
* must provide value

- ◯ Yes , I have checked all the boxes myself and I agree to take part in the study
- ◯ No

reset

## Please sign

**...to show your agreement to participate in this study**
* must provide value

Add signature

Click "add signature" and use your mouse or finger if using a touch screen to draw your signature

**You need to select all the boxes in the consent form to proceed.** However, if you cannot agree with them all, you don't have to do anything further, simply close this browser window.

Have a good day!

Vainre et al. 2021 SWELL Protocol Supplementary Material 2: Consent form

# Thank you!

When you click submit, we'll send you an e-mail to make sure we have your contact details right. Please give a shout if you didn't receive the e-mail (not even to your junk mail), otherwise we may not be able to contact you again.

**Now click submit!**

**Just in case you might find it helpful, here's a guide for getting mental health support.**

Attachment: Swell_MentalHealthResources.pdf (0.13 MB)

For peer review only

BMJ Open

# Protocol for the Work Engagement and Well-being Study (SWELL): A randomised controlled feasibility trial evaluating the effects of mindfulness versus light physical exercise at work



| | |
|---|---|
| Journal: | *BMJ Open* |
| Manuscript ID | bmjopen-2021-050951.R1 |
| Article Type: | Protocol |
| Date Submitted by the Author: | 04-Feb-2022 |
| Complete List of Authors: | Vainre, Maris; University of Cambridge<br>Galante, Julieta; University of Cambridge, Department of Psychiatry<br>Watson, Peter; University of Cambridge, MRC Cognition and Brain Sciences Unit<br>Dalgleish, Tim; Medical Research Council Cognition and Brain Sciences Unit<br>Hitchcock, Caitlin; University of Cambridge, MRC Cognition and Brain Sciences Unit; The University of Melbourne Melbourne School of Psychological Sciences |
| <b>Primary Subject Heading</b>: | Mental health |
| Secondary Subject Heading: | Evidence based practice, Public health |
| Keywords: | PUBLIC HEALTH, PREVENTIVE MEDICINE, OCCUPATIONAL & INDUSTRIAL MEDICINE |
| | |



SCHOLARONE™
Manuscripts

# Protocol for the Work Engagement and Well-being Study (SWELL): A randomised controlled feasibility trial evaluating the effects of mindfulness versus light physical exercise at work

Ms Maris Vainre, maris.vainre@mrc-cbu.cam.ac.uk, MRC Cognition and Brain Sciences Unit, University of Cambridge, Cambridge, UK

Dr Julieta Galante, Department of Psychiatry, University of Cambridge, Cambridge, UK

Dr Peter Watson, MRC Cognition and Brain Sciences Unit, University of Cambridge, Cambridge, UK

Prof Tim Dalgleish, MRC Cognition and Brain Sciences Unit, University of Cambridge, Cambridge, UK, Cambridgeshire and Peterborough NHS Foundation Trust, Cambridgeshire and Peterborough, UK

Dr Caitlin Hitchcock, MRC Cognition and Brain Sciences Unit, University of Cambridge, Cambridge, UK and Melbourne School of Psychological Sciences, The University of Melbourne

Keywords: mindfulness, work, well-being, productivity, randomised controlled trial

For peer review only

# ABSTRACT

## Introduction

Mental ill health is a major cause of disability. Workplaces are attractive for preventative interventions since most adults work; meanwhile, employers are interested in improving employees' well-being and productivity. Mindfulness-based programmes are increasingly popular in occupational settings. However, there is inconsistent evidence whether mindfulness interventions improve work performance and how effective mindfulness-based programmes are, compared to other interventions, in preventing mental ill health.

## Methods and analysis

In this online randomised controlled feasibility trial, an anticipated 240 employees will be randomised to either a 4-week light physical exercise course or a mindfulness course of the same duration (1:1 allocation). The primary outcome is work performance, measured using the Work Role Functioning Questionnaire. We aim to evaluate the acceptability, feasibility, and procedural uncertainties of a randomised controlled trial in a workplace, calculate an effect size estimate to inform power calculations for a larger trial, and explore whether improved executive function and/or enhanced mental health could be potential mechanisms underlying the effect of mindfulness on work performance. Outcomes will be collected at baseline, post-intervention and 12-week follow-up.

## Ethics and dissemination

Approval has been obtained from Cambridge Psychology Research Ethics Committee (PRE.2020.072). Results will be published in peer-reviewed journals. A lay summary will be disseminated to a wider audience including participating employers.

## Registration details

Clinicaltrials.gov: NCT04631302

# ARTICLE SUMMARY

Strengths and limitations of this study:

- A randomised trial to lay the foundations to investigate the mechanisms of mindfulness intervention underlying effects on work performance.
- The study employs a range of outcome measures, including self-reported measures and cognitive functioning tasks.

For peer review only

- This feasibility trial is not powered to detect significant effects, but rather to estimate effect size to inform design of a larger later-stage trial.
- Several feasibility outcomes will be collected to inform a later-stage trial.

For peer review only

# INTRODUCTION

## Background and rationale

Mental illness is a major cause of disability worldwide[1]. Much of the adult population is employed and spends 28% of their waking hours doing paid work[2,3]. The occupational environment is therefore an opportune location for preventative mental health interventions.

Poor mental health is responsible for 44% of work-related episodes of ill health[4] and according to conservative estimates, is thought to cost the United Kingdom's (UK) economy £45 billion annually[5] or 2% of UK's Gross Domestic Product. To reduce this burden, a growing number of employers provide programmes to improve well-being and work performance.

Mindfulness-based programmes (MBPs) are increasingly popular in occupational settings. Mindfulness is typically defined as "the awareness that emerges through paying attention on purpose, in the present moment, and nonjudgmentally to the unfolding of experience moment by moment"[6]. Practicing such awareness is linked to reduction in symptoms of anxiety, depression, and stress in community populations[7,8]. There is also evidence that mindfulness training could improve overall well-being[8,9], life satisfaction[10], and quality of life[7].

Mindfulness practice may yield workplace benefits beyond emotional well-being. It has been proposed that mindfulness improves work performance[11] and reduces the negative effects of multitasking[12]. Yet, there is little evidence to support these claims. A recent meta-analysis concluded that work performance was rarely assessed in trials investigating the outcomes of MBPs. When work performance was measured, wide-ranging operational definitions were used: e.g., engagement[13–17], motivation[14], absenteeism[18] and presenteeism[16–18], rate of errors[19] and burnout[20]. Thus, estimating an overall effect is difficult[21,22]. Methods for measuring performance in higher education have less variability, yet there is no clear indication that offering mindfulness training to university students improves their academic performance[23].

The mechanisms underlying any effect of mindfulness on work performance are also yet to be determined while two mechanistic pathways stand out that could explain such an effect of MBPs. First, positive effects of MBPs on mental well-being are well-established[7–9], and mental well-being is linked to better work performance[24,25]. Conversely, mental health problems decrease employees' performance[26–28], particularly if these problems are poorly managed[29]. However, an indirect effect of MBPs on workplace performance via improved mental well-being has yet to be evaluated.

A second potential mechanism could be an improved cognitive control over mental activity, which allows one to prioritise current task-relevant goals[30,31]. There are three potential

For peer review only

facets of cognitive control that may be improved by MBPs: (a) shifting, that is, the ability to switch between multiple tasks; (b) updating, or the ability to frequently refresh information in working memory to ensure a currently relevant record of information; and (c) inhibition: deliberately hindering dominant or automatic responses that are irrelevant to the task at hand[32]. Improved cognitive control, in turn, may lead to better performance on workplace tasks[11,33].

Mindfulness has been shown to have a small effect on cognitive control[34,35]. A recent meta-analysis analysing outcomes of randomised controlled trials (RCTs) measuring the effects of cognitive control in MBPs for healthy participants found a small overall effect of Hedge's $g$=0.2[35]. However, we know little about how these changes in cognitive control manifest in the workplace[21]. While mindfulness may improve performance on tasks closely related to the practice, such as counting breaths[36], it may not extend to other tasks, such as those completed at work[37].

Furthermore, to date, research has primarily focussed on the impact of mindfulness on cognitive control over emotionally neutral information. Yet, much of the everyday mental activity that we seek to regulate is emotionally positive or negative[38,39]. In the two meta-analysis of MBPs' effects on cognitive control published to date[34,35], only one identified study used emotional stimuli within an cognitive control task. This study reported a null-effect of meditation on cognitive control measured via an attention network test when comparing negative and neutral conditions[40]. It is important to note that this study[40] was likely underpowered.

At work, it is arguably beneficial to inhibit emotional thoughts (e.g., worrying about a recent argument with your spouse) that are irrelevant to the task at hand (e.g., writing a report). A reduced ability to inhibit internal emotional stimuli may interfere with our ability to maintain focus on workplace tasks. There is evidence that emotional stimuli inhibit cognitive control, when measured using the Stop-Signal Task[41–43]. As mindful meditation trains the ability to move away from thoughts and images of negative emotional valence, practicing mindfulness may enhance cognitive control over emotional mental events[44]. It is therefore important to determine whether MBPs improve workplace performance via enhancement of cognitive control skills such as the ability to move away from negative stimuli (e.g., worries about task performance) or to decentre from negative mental content[45,46] and refocus attention on the task at hand[22].

Understanding the mechanisms underlying effects of MBPs on work performance would (a) help to design more targeted interventions, (b) improve our attempts to assess MBPs, by designing and selecting more stringent outcome measures and control interventions and (c) inform an understanding of for whom MBPs may be most effective, and in which context[47].

For peer review only

# Objectives

Current literature suggests that MBPs could improve work performance through increased mental well-being and/or cognitive control over emotional material. In order to test this, we need to control for one of the two pathways. Both the MBP and light exercise have been shown to reduce stress, depression and anxiety[48–50], however, only mindfulness is expected to improve cognitive control skills. We chose light exercises as a condition to help to distinguish between the different pathways through which work performance may improve.

A definitive randomised controlled trial is needed to evaluate these potential mechanisms. However, methodological uncertainties and questions of acceptability and feasibility need clarification to inform the design of such a trial[51–53]. We aim to conduct a feasibility trial to clarify these uncertainties and complete a preliminary investigation of the relationships between mindfulness training, workplace performance and the proposed mechanisms of action.

This feasibility trial will:

1. Estimate the between-groups effect size for the effect of mindfulness, relative to a light exercise control condition, on our primary outcome of work performance, in order to inform power calculation for a larger trial;
2. Explore whether improved cognitive control and/or enhanced mental health could be potential mechanisms underlying the effect of mindfulness on work performance;
3. Assess the acceptability of the interventions and the study design by monitoring recruitment, retention, and adherence to the course;
4. Determine procedural feasibility of a later stage trial by evaluating the willingness of the participants to be randomised and other practical implications of running a randomised controlled trial at a workplace.

# METHODS

This protocol follows the guidelines for RCTs set by the SPIRIT 2013 statement[54] (SPIRIT checklist in Supplementary File 1). The study's prospective registration number at clinicaltrials.gov is NCT04631302. Initial consent taking started in November 2020. Participants, irrespective of the time they consent, received access to baseline measures from the 23rd February 2021. Data collection will finish by the end of February 2022.

# Study design

We will conduct a participant-level RCT. Employees will be randomly allocated in a 1:1 ratio to either of two parallel groups.

# Eligibility criteria

For peer review only - http://bmjopen.bmj.com/site/about/guidelines.xhtml

Eligibility to participate in this study will be self-reported. The employers who have agreed to participate in the study are local councils or education providers or trade either in the publishing, electronics, or construction industry with employees in a variety of roles, mostly in desk-based occupations. Individuals can participate if they work for the employers taking part in this trial, are based in the UK, and are not currently on a long-term leave. We will recommend that a participant chooses not to join the study if they:

- are currently suffering from severe periods of anxiety, depression or hypomania/mania;
- are experiencing other severe mental illnesses;
- have had a recent bereavement or major loss;
- have already completed a mindfulness course or have meditated more than 10 hours in the past 10 years.

# Intervention condition: *Be Mindful* mindfulness-based programme

Participants in the Mindfulness condition will complete the *Be Mindful* pre-recorded online course by Wellmind Media. It incorporates elements from Mindfulness-Based Stress Reduction[55] and Mindfulness-Based Cognitive Therapy[56]. Course materials and instructional videos are accessed through a website (http://www.bemindfulonline.com).

The four-week course consists of 10 sessions led by two teachers, one female, one male. Participants are taught to use formal meditations (focusing attention on the practice of meditation) as well as informal mindfulness techniques, such as mindful walking and mindful eating. Daily homework includes a formal mediation practice with the assistance of video/audio recordings (up to 30 minutes), and one or two informal exercises per day (see Table 1 for an overview). Every week, participants receive e-mails motivating them to practice and informing them when the next module is available. As this is a feasibility examination for a pragmatic trial, no modifications to the procedures to maintain adherence to the intervention will be implemented.

# Control condition: Light physical exercise

The four-week control condition involves light exercises aimed at increasing mobility, reducing stiffness, improving blood circulation, and avoiding pain or repetitive strain injuries that may result from sedentary or repetitive tasks common in office environments. The pre-recorded exercises will include whole-body slightly aerobic exercises such as rotation of joints and stretching. The course was developed by JG, a public health doctor, together with an expert in body posture.

The control condition course is designed to match the intervention condition in overall time commitment, and the frequency of interaction with the participant (see Table 1). It replicates

For peer review only

the encouraged use of short breaks throughout the workday to focus on well-being, as in the intervention condition.

Table 1. Comparison of the intervention and control group

| Condition | Intervention: Be Mindful | Control: Light physical exercise |
|---|---|---|
| **Number of sessions in total** | 10 | 28 |
| **Online coursework frequency** | Twice weekly | Daily |
| **Typical session and its length** | Self-paced. Includes videos (average of 3-4 minutes in total per session), self-reflection exercises and brief reading tasks. | Videos of 10-13 minutes. |
| **Homework frequency** | Daily | Daily |
| **Typical assignment** | A formal meditation (10-30 minutes) and shorter task such as journaling or noticing. The frequency of the latter varies from daily to once a week. | Using the exercises while taking brief breaks during the day. |
| **Reminders to encourage practicing** | 4 times a week | 4 times a week |

# Data collection

Data collection will take place at baseline (T0), after the courses finish (T1) and 12 weeks after completing the courses (T3) (see **Error! Reference source not found.**). Additionally, a brief questionnaire will be sent to the participants each workday. Data collected at T1 will be considered as the primary end-point of interest.

Figure 1 should be inserted here

# Outcomes

## Feasibility, acceptability, and procedural outcomes

To determine feasibility of a later-stage trial, we will examine descriptive statistics to:

1. Estimate between-condition effect sizes:
    a. for the primary outcome, to inform a power calculation for a later-stage trial;
    b. for the cognitive control outcomes, to determine suitability of these measures to index mechanisms of interest.
2. Determine feasibility of running a later-stage trial by monitoring recruitment (the percentage of employees who consent into the study), retention, including timing of measurements (by indexing percentage of participants completing each time point),

For peer review only

and potential contamination issues, most notably, measuring the extent to which participants talked about their course with participants from the other arm;

3. Acceptability of interventions by indexing which course the participants would have preferred to be randomised to, their regularity in engaging in exercise and mindfulness, and intervention dose, notably the percentage of course materials attempted;
4. Procedural uncertainties, for example, by exploring the suitability of our primary measure in indexing our primary outcome. To this end, we have introduced several work-related outcomes (see Secondary Outcomes);
5. Potential covariates influencing key outcomes which may need to be considered in design of the later-stage trial, including:
   a. participant mental and physical health at baseline;
   b. importance of job to participants' identity at baseline.

## Primary outcome: Work performance

Work performance will be measured by using the 25-item Work Role Functioning Questionnaire's[57] updated version[58], to capture perceived difficulties in meeting work demands. Items are rated on a 5-point scale ('difficult all the time' to 'difficult none of the time'), with higher scores indicating better functioning. A 6th option denotes 'does not apply to my job'. The questionnaire has not been validated in English. Validations completed in Dutch[58], Spanish[59], and Norwegian[60] have shown good internal consistency (Cronbach alphas 0.7-0.9)[58–60], and test-retest reliability (ICC=0.66, 95% CI: 0.54-0.76 for the total score)[58]. The WRFQ features four subscales: work scheduling and output demands (α=0.92), physical demands (α=0.92), mental and social demands (α=0.91), and flexibility demands (α=0.96)[58]. The WRFQ has been shown to possess decent convergent validity, correlating with similar measures including the Utrecht Work Engagement Scale[61] (r=0.304)[58], Work Ability Index[62] (r=0.468)[58]. The primary endpoint in this trial will be the post-intervention measurement. Feasibility of using the 12-week follow-up as the main outcome end-point in the later-stage trial, will be assessed. We recognise that effects at 12 weeks may not be sustained longer term. Retention at 12 weeks will help to plan the sample size for a larger trial which could also then measure outcomes longer term.

## Secondary outcomes

### *Work-related outcomes*

The participants are asked to report if they have health conditions that affect their ability to work, with options to pick one or several of the following: physical health problems, mental health problems, other health problems, no problems or prefer not to say. If a participant selects one of the first three options (i.e., they have had problems), they will be asked to briefly describe these problems.

Those who self-report experiencing mental or physical health problems in the item described above will be asked to fill in the Work and Social Adjustment Scale[63]. The scale is widely

For peer review only

used in the NHS psychology services in England and has good internal consistency, α=0.82[64].

To get a better understanding of daily fluctuations that may occur in work engagement, participants will be asked to complete a 5-item version of the Work Role Functioning Questionnaire[65] each workday afternoon. Items are rated the same as in the full Work Role Functioning Questionnaire.

## *Cognitive control mechanisms*

Two online computerised cognitive tasks will be used, to index our potential executive function mechanisms of interest. Affective cognitive control will be assessed using the Emotional Stop-Signal Task[66]. At the beginning of each trial within the task, a negative or a neutral image appears, followed by a go-signal (left or right arrow). Participants need to respond with a corresponding key-press. On a minority of trials (20%), the go-signal is followed by a stop-signal (upwards arrow) in which a go-response is required to be inhibited. Reaction times (in both, go- and stop-trials), response accuracy (failure or success in inhibiting response) and variability in reaction time throughout the task (a proxy for the ability to overcome errors) will be measured. The main outcome of interest is the response time in stop-trials.

Participant's ability to track dynamic changes in their environment and alter their response strategies will be measured using an affective modification of the probabilistic reversal learning task[67]. The task will consist of 6 phases, 3 for the neutral and 3 for the negative condition. Each trial will begin with a negative or a neural image from the International Affective Picture System (IAPS)[68]. Next, pairs of stimuli (A-B or C-D) will be presented. Participants must select a stimulus with a key-press. In each pair, one of the stimuli is more likely to be rewarded (e.g., selecting A or C is reinforced on 80% of trials). Feedback is presented after each response. Through trial-and-error, participants learn which stimuli are more frequently rewarded. After a certain number of trials (a phase), the contingency of reinforcement switches. In Phase 2, the other stimulus in the pair is more frequently reinforced (e.g., instead of A, B is now reinforced on 80% of trials). In Phase 3, the reinforcement is switched again. Reaction times and response accuracy (i.e., selecting the reinforced stimulus) will be recorded. The main outcome of interest will be changes in learning performance indexed via the proportion of correct responses.

## *Other outcomes of interest*

### Well-being

Subjective mental well-being will be measured with the Short Warwick-Edinburgh Mental Well-being Scale (SWEMWBS), a 7-item questionnaire designed to capture a broad concept of well-being[69]. In SWEMWBS, items are scored on a scale of 1-5 ('none of the time'…'all of

For peer review only - http://bmjopen.bmj.com/site/about/guidelines.xhtml

the time'), with higher scores suggesting better mental well-being. The SWEMWBS internal consistency was α=0.84 in a study in the UK general population (n=27,169)[70].

### Stress

The Perceived Stress Scale (PSS) measures the extent to which the individual has perceived events as uncontrollable and overwhelming. The PSS consists of 10 items, answered on a scale of 0-4, higher scores indicate higher stress levels. The PSS possesses good internal consistency, α=0.84-0.86[71].

### Depression

The Patient Health Questionnaire (PHQ-9)[72] is used to assess depression. It consists of 9 items answered using a scale from 0-3, and a further item asking about the level of difficulty associated with any checked off items. Total scores range from 0-27 with cut-off points for depression at 5, 10, 15 and 20 for mild, moderate, moderately severe and severe depression, respectively[72]. A PHQ-9 score of at least 10 has been found to have 88% sensitivity and 88% specificity for major depression[72].

### Anxiety

The General Anxiety Disorder 7-item Scale (GAD-7)[73] assesses anxiety and has good reliability and validity[74]. The items are answered using a scale from 0-3, yielding total scores between 0 and 21 with cut-offs at 5, 10 and 15 for mild, moderate and severe anxiety, respectively[73]. The scale's internal consistency is α=.92. A total score of 10 has a 89% sensitivity and 82% specificity for generalised anxiety disorder[73].

## *Mindfulness-related outcomes*

The following will be administered to ensure that the MBP does increase mindfulness more than the control condition.

### Decentering

The Experiences Questionnaire (EQ)[45] is an 11-item measure of decentering. The items were generated to represent the changes believed to occur due to mindfulness practice, including the extent to which one's self-identity depends on one's thoughts, nonreactivity to negative experiences, and self-compassion. Statements are rated on a 5-point scale ('never' to 'all the time'), with higher scores reflecting higher levels of decentering. The scale's internal consistency is α=.81–.84[45].

### Mindfulness

Mindful Attention Awareness Scale (MAAS)[75] is a self-report questionnaire consisting of 15 items designed to assess a core characteristic of mindfulness – a receptive state of mind in which attention simply observes what is taking place. Items are rated using a 6-point Likert

For peer review only

scale ('almost always' to 'almost never'), with higher scores indicating more mindfulness. The internal consistency of MAAS is α=.87[75].

# Sample size

One of the procedural uncertainties limiting the design of a fully-powered trial is the size of the effect on the main outcome in interest. As a traditional power calculation is unfeasible given the lack of previous data, we seek to determine the likely effect size in this study, to inform a later-phase trial.

We aim to recruit 240 participants. A fully online design may cause a high loss to follow-up; a systematic review of internet-based RCTs found the average attrition rate to be 47% at post-intervention[76]. Based on this, we have selected a sample size which we anticipate will yield complete data for 128 participants at our primary end-point of post-intervention (64 per arm) and 68 participants at follow-up (34 per arm). In clinical research with lower attrition rates, feasibility trials tend to recruit 36 participants[77]. Considering high risk of attrition and the considerable uncertainties regarding the feasibility of the trial, we estimate that our sample size is optimal to examine the feasibility of procedures and provide a reliable estimate of effect size.

# Study procedures

## Recruitment

Employers who have agreed to collaborate in the research project, have taken an active role in shaping the recruitment process to their organisational customs. The invitation, sent via web-based communication services used by the employer, will have a link to the participant information sheet and consent form.

## Inducements for participation

There will be no inducements for completing either of the interventions. As a token of appreciation for completing the study assessments, participants will be given £10 at post-intervention and £15 at follow-up time points in the form of retail vouchers.

## Randomisation procedure

After the participants have completed all baseline measurements, they will be randomised to either the mindfulness or the light physical exercise arm, stratified by employer. The randomisation process will be automated using REDCap, a platform for questionnaire data collection[78,79]. The allocation tables were generated with randomizeR package[80] in R using randomised permuted block randomisation with pre-specified seeds for reproducibility. The code is available at GitHub[81].

For peer review only

Participants will not be blind to their allocation. The primary analysis will be completed by a statistician (PW) blind to intervention allocation.

# Public involvement

The study's design has been formed by feedback from the employers participating in the study, including the perceived utility of the interventions, recruitment procedure and its timing, study materials, incentives for participation and outcomes. Changes to the initial design were proposed, some of which were implemented (e.g., offering vouchers rather than cash; channels and timing for recruitment).

# Statistical methods

Central tendencies, dispersion, and data missingness will be reported for all time points. At baseline, descriptive statistics will be presented overall and by group allocation. At following timepoints, outcomes will be reported by group.

Any significance testing, though not the focus of this trial, will follow the intention-to-treat principle. A key limitation of feasibility trials such as this is that adequate power is not obtained to detect statistical significance. For the primary outcome, a linear multiple regression model will compare the WRFQ total score between trial arms at post-intervention, adjusted for baseline WRFQ and employer. Multiple imputation will be used to account for missing data. Further exploratory analysis will employ the same approach for other outcome measures at post-intervention and 12-week follow-up. Mediation analysis techniques will be employed to assess the suggested mechanistic pathways. Effect sizes obtained in these analyses will be used to inform a potential later-stage trial and are the focus of this trial, rather than statistical significance.

For the secondary outcomes, mixed model repeated measures analysis will be performed using the daily monitoring of work performance to study changes between arms during the intervention. The analysis will also compare different work performance measures. Again, the focus of this trial is on obtaining an estimate of likely effect sizes, rather than statistical significance.

# Data monitoring and adverse events

An Independent Study Steering group has been established to monitor data and advise the conduct of the study to ensure participant safety and integrity of research. We have established the following safeguards[82,83]:

1. Participants are made aware they may request a consultation with a clinical psychologist if they feel uncomfortable with the study or experience discomfort they associate with the interventions.

For peer review only

2. Where participants' responses to PHQ9 (depression) or GAD7 (anxiety) are above clinical cut-off scores (≥20 and ≥15, respectively), a warning is automatically sent to the researcher. For PHQ9, the alert is also triggered when the participant score is >0 on the self-harm item. The researcher (MV) will then consult the clinical psychologist who will contact the participant.

3. Participants wishing to leave the study will be encouraged to let the study team know why they have chosen to do so.

Any adverse events discovered through the mechanisms listed above will be discussed with the Independent Study Steering group who may decide whether they are attributable to the interventions (i.e., adverse effects) and any subsequent course of action.

# ETHICS AND DISSEMINATION

The trial has received approval from the Cambridge Psychology Research Ethics Committee PRE.2020.072.

## Consent

The consent form states eligibility criteria and the circumstances in which we recommend not to participate in the study. Participants are invited to join virtual information sessions or e-mail the study team should they have any questions.

Information about accessing mental health support services is made available to anyone visiting the participation information website and e-mailed to those who consent to the study. Only those who consent to participate will receive the link to baseline measurements.

## Data management

Data will be collected and curated using the Research Electronic Data Capture (REDCap)[78,79], the Cohort Management System (CMS) and JATOS[84]. Anonymised data will be shared for research purposes upon request, in line with open science principles. All personally identifiable data will be separated from study data and stored on separate encrypted servers.

## Dissemination policy

Findings will be submitted to peer-review journals. Authorship in publications will be based on the International Committee of Medical Journal Editors' criteria. We will also send a lay summary of the results to the participating employers and participants.

For peer review only

# ACKNOWLEDGEMENTS

The study team would like to thank Becky Gilbert, Camilla Nord, Marc Bennett, Rachel Knight, and Tierney Lee for their role in designing the cognitive tasks, Christina Haag for her consultations on planned analyses, and Markus Hausammann for substantially contributing to the design of the study logo.


# CONTRIBUTORS

MV, CH, JG and TD developed the idea for the trial and applied for funding. MV, CH and JG drafted the protocol which was then revised through discussions with TD, the Emotion study group at MRC CBU and the participating employers. The analysis plan was devised by MV, CH, JG and PW. MV is the lead researcher. CH and JG are supervising the research. The trial is sponsored by University of Cambridge.


# FUNDING STATEMENT

This study is supported by MRC Cognition and Brain Sciences Unit, MV is supported by Kristjan Jaak degree studies abroad scholarship by Estonia's Education and Youth Board, JG by the National Institute for Health Research (NIHR) Applied Research Collaboration East of England, CH by the Economic and Social Research Council and TD by the UK Medical Research Council (Grant reference: SUAG/043 G101400) and Wellcome (Grant reference: 104908/Z/14/Z, 107496/Z/15/Z). The views expressed are those of the authors and not necessarily those of the National Health Service, the NIHR or the Department of Health and Social Care.


# COMPETING INTERESTS' STATEMENT

None declared.

For peer review only

# REFERENCES

1 James SL, Abate D, Abate KH, *et al.* Global, regional, and national incidence, prevalence, and years lived with disability for 354 diseases and injuries for 195 countries and territories, 1990–2017: a systematic analysis for the Global Burden of Disease Study 2017. *The Lancet* 2018;**392**:1789–858. doi:10.1016/S0140-6736(18)32279-7

2 OECD.Stat. Employment: Time spent in paid and unpaid work, by sex. https://stats.oecd.org/index.aspx?queryid=54757

3 Office for National Statistics. Awake time per day in the United Kingdom (UK) in 2014, by age and gender. Statista. 2014.https://www.statista.com/statistics/326907/awake-time-per-day-uk-by-age-and-gender/ (accessed 15 Jul 2020).

4 Health and Safety Executive. Work-related stress, anxiety or depression statistics in Great Britain, 2019. Health and Safety Executive 2019. https://www.hse.gov.uk/statistics/causdis/stress.pdf (accessed 12 May 2020).

5 Deloitte UK. Mental Health and Employers: Refreshing the case for investment. Deloitte 2020. https://www2.deloitte.com/content/dam/Deloitte/uk/Documents/consultancy/deloitte-uk-mental-health-and-employers.pdf

6 Kabat-Zinn J. Mindfulness-based interventions in context: Past, present, and future. *Clin Psychol Sci Pract* 2003;**10**:144–56. doi:10.1093/clipsy/bpg016

7 Khoury B, Sharma M, Rush SE, *et al.* Mindfulness-based stress reduction for healthy individuals: A meta-analysis. *J Psychosom Res* 2015;**78**:519–28. doi:10.1016/j.jpsychores.2015.03.009

8 Galante J, Friedrich C, Dawson AF, *et al.* Mindfulness-based programmes for mental health promotion in adults in nonclinical settings: A systematic review and meta-analysis of randomised controlled trials. *PLOS Med* 2021;**18**:e1003481. doi:10.1371/journal.pmed.1003481

9 Spijkerman MPJ, Pots WTM, Bohlmeijer ET. Effectiveness of online mindfulness-based interventions in improving mental health: A review and meta-analysis of randomised controlled trials. *Clin Psychol Rev* 2016;**45**:102–14. doi:10.1016/j.cpr.2016.03.009

10 Shapiro SL, Astin JA, Bishop SR, *et al.* Mindfulness-Based Stress Reduction for Health Care Professionals: Results From a Randomized Trial. *Int J Stress Manag* 2005;**12**:164–76. doi:10.1037/1072-5245.12.2.164

11 Dane E. Paying Attention to Mindfulness and Its Effects on Task Performance in the Workplace. *J Manag* 2011;**37**:997–1018. doi:10.1177/0149206310367948

12 Shapiro SL, Wang MC, Peltason EH. What is mindfulness, and why should organizations care about it? In: Reb J, Atkins PWB, eds. *Mindfulness in Organizations*. Cambridge: : Cambridge University Press 2015. 17–41. doi:10.1017/CBO9781107587793.004

For peer review only

13 Aikens KA, Astin J, Pelletier KR, *et al.* Mindfulness Goes to Work: Impact of an Online Workplace Intervention. *J Occup Environ Med* 2014;**56**:721–31.

14 Wilson DM. *Effects of mindfulness-based art processing (MBAP) on the well-being and job performance of working adults: Evaluating a novel intervention*. 2012.

15 Klatt M, Steinberg B, Duchemin A-M. Mindfulness in Motion (MIM): An Onsite Mindfulness Based Intervention (MBI) for Chronically High Stress Work Environments to Increase Resiliency and Work Engagement. *JoVE J Vis Exp* 2015;:e52359. doi:10.3791/52359

16 van Berkel J, Boot CRL, Proper KI, *et al.* Effectiveness of a Worksite Mindfulness-Related Multi-Component Health Promotion Intervention on Work Engagement and Mental Health: Results of a Randomized Controlled Trial. *PLoS ONE* 2014;**9**:e84118. doi:10.1371/journal.pone.0084118

17 van Dongen JM, van Berkel J, Boot CRL, *et al.* Long-Term Cost-Effectiveness and Return-on-Investment of a Mindfulness-Based Worksite Intervention: Results of a Randomized Controlled Trial. *J Occup Environ Med* 2016;**58**:550–60. doi:10.1097/JOM.0000000000000736

18 Bartlett L, Lovell P, Otahal P, *et al.* Acceptability, Feasibility, and Efficacy of a Workplace Mindfulness Program for Public Sector Employees: a Pilot Randomized Controlled Trial with Informant Reports. *Mindfulness* 2017;**8**:639–54. doi:10.1007/s12671-016-0643-4

19 Verweij H, van Ravesteijn H, van Hooff MLM, *et al.* Mindfulness-Based Stress Reduction for Residents: A Randomized Controlled Trial. *J Gen Intern Med* 2018;**33**:429–36. doi:10.1007/s11606-017-4249-x

20 Luken M, Sammons A. Systematic Review of Mindfulness Practice for Reducing Job Burnout. *Am J Occup Ther* 2016;**70**:7002250020p1–10. doi:10.5014/ajot.2016.016956

21 Bartlett L, Martin A, Neil AL, *et al.* A systematic review and meta-analysis of workplace mindfulness training randomized controlled trials. *J Occup Health Psychol* 2019;**24**:108–26. doi:10.1037/ocp0000146

22 Jamieson SD, Tuckey MR. Mindfulness interventions in the workplace: A critique of the current state of the literature. *J Occup Health Psychol* 2017;**22**:180–93. doi:10.1037/ocp0000048

23 Dawson AF, Brown WW, Anderson J, *et al.* Mindfulness-Based Interventions for University Students: A Systematic Review and Meta-Analysis of Randomised Controlled Trials. *Appl Psychol Health Well-Being* 2019;**12**.https://onlinelibrary.wiley.com/doi/abs/10.1111/aphw.12188 (accessed 24 Feb 2020).

24 García-Buades ME, Peiró JM, Montañez-Juan MI, *et al.* Happy-Productive Teams and Work Units: A Systematic Review of the 'Happy-Productive Worker Thesis.' *Int J Environ Res Public Health* 2020;**17**. doi:10.3390/ijerph17010069

For peer review only

25 Montano D, Reeske A, Franke F, *et al.* Leadership, followers' mental health and job performance in organizations: A comprehensive meta-analysis from an occupational health perspective. *J Organ Behav* 2017;**38**:327–50. doi:10.1002/job.2124

26 Alonso J, Petukhova M, Vilagut G, *et al.* Days out of role due to common physical and mental conditions: results from the WHO World Mental Health surveys. *Mol Psychiatry* Published Online First: 2011. doi:10.1038/mp.2010.101

27 Collins JJ, Baase CM, Sharda CE, *et al.* The Assessment of Chronic Health Conditions on Work Performance, Absence, and Total Economic Impact for Employers. *J Occup Environ Med* 2005;**47**:547–57. doi:10.1097/01.jom.0000166864.58664.29

28 Stewart WF, Ricci JA, Chee E, *et al.* Cost of Lost Productive Work Time Among US Workers With Depression. *JAMA* 2003;**289**:3135–44. doi:10.1001/jama.289.23.3135

29 Rost K, Smith JL, Dickinson M. The Effect of Improving Primary Care Depression Management on Employee Absenteeism and Productivity A Randomized Trial. *Med Care* 2004;**42**:1202–10.

30 Schweizer S, Satpute AB, Atzil S, *et al.* The impact of affective information on working memory: A pair of meta-analytic reviews of behavioral and neuroimaging evidence. *Psychol Bull* 2019;**145**:566–609. doi:10.1037/bul0000193

31 Ionescu T. Exploring the nature of cognitive flexibility. *New Ideas Psychol* 2012;**30**:190–200. doi:10.1016/j.newideapsych.2011.11.001

32 Miyake A, Friedman NP, Emerson MJ, *et al.* The Unity and Diversity of Executive Functions and Their Contributions to Complex "Frontal Lobe" Tasks: A Latent Variable Analysis. *Cognit Psychol* 2000;**41**:49–100. doi:10.1006/cogp.1999.0734

33 Chaskalson M. *The mindful workplace: developing resilient individuals and resonant organizations with MBSR*. Chichester, West Sussex; Malden, MA: : Wiley-Blackwell 2011.

34 Cásedas L, Pirruccio V, Vadillo MA, *et al.* Does Mindfulness Meditation Training Enhance Executive Control? A Systematic Review and Meta-Analysis of Randomized Controlled Trials in Adults. *Mindfulness* 2020;**11**:411–24. doi:10.1007/s12671-019-01279-4

35 Yakobi O, Smilek D, Danckert J. The Effects of Mindfulness Meditation on Attention, Executive Control and Working Memory in Healthy Adults: A Meta-analysis of Randomized Controlled Trials. *Cogn Ther Res* Published Online First: 4 January 2021. doi:10.1007/s10608-020-10177-2

36 Levinson DB, Stoll EL, Kindy SD, *et al.* A mind you can count on: Validating breath counting as a behavioral measure of mindfulness. *Front Psychol* 2014;**5**:1–10. doi:10.3389/fpsyg.2014.01202

37 Simons DJ, Boot WR, Charness N, *et al.* Do "brain-training" programs work? *Psychol Sci Public Interest Suppl* 2016;**17**:103–86. doi:10.1177/1529100616661983

For peer review only

38 Killingsworth MA, Gilbert DT. A Wandering Mind Is an Unhappy Mind. *Science* 2010;**330**:932–932. doi:10.1126/science.1192439

39 Kragel PA, Knodt AR, Hariri AR, *et al.* Decoding Spontaneous Emotional States in the Human Brain. *PLOS Biol* 2016;**14**:e2000106. doi:10.1371/journal.pbio.2000106

40 Ainsworth B, Eddershaw R, Meron D, *et al.* The effect of focused attention and open monitoring meditation on attention network function in healthy volunteers. *Psychiatry Res* 2013;**210**:1226–31. doi:10.1016/j.psychres.2013.09.002

41 Verbruggen F, De Houwer J. Do emotional stimuli interfere with response inhibition? Evidence from the stop signal paradigm. *Cogn Emot* 2007;**21**:391–403. doi:10.1080/02699930600625081

42 Kalanthroff E, Cohen N, Henik A. Stop feeling: inhibition of emotional interference following stop-signal trials. *Front Hum Neurosci* 2013;**7**. doi:10.3389/fnhum.2013.00078

43 Herbert C, Sütterlin S. Response Inhibition and Memory Retrieval of Emotional Target Words: Evidence from an Emotional Stop-Signal Task. *J Behav Brain Sci* 2011;**1**. doi:10.4236/jbbs.2011.13020

44 Crane RS, Brewer J, Feldman C, *et al.* What defines mindfulness-based programs? The warp and the weft. *Psychol Med* 2017;**47**:990–9. doi:10.1017/S0033291716003317

45 Fresco DM, Moore MT, van Dulmen MHM, *et al.* Initial Psychometric Properties of the Experiences Questionnaire: Validation of a Self-Report Measure of Decentering. *Behav Ther* 2007;**38**:234–46. doi:10.1016/j.beth.2006.08.003

46 Safran JD, Segal ZV. *Interpersonal process in cognitive therapy*. 1st softcover ed. Northvale, N.J: : Jason Aronson 1996.

47 Nielsen L, Riddle M, King JW, *et al.* The NIH Science of Behavior Change Program: Transforming the science through a focus on mechanisms of change. *Behav Res Ther* 2018;**101**:3–11. doi:10.1016/j.brat.2017.07.002

48 Galante J, Bekkers M-J, Mitchell C, *et al.* Loving-Kindness Meditation Effects on Well-Being and Altruism: A Mixed-Methods Online RCT. *Appl Psychol Health Well-Being* 2016;**8**:322–50. doi:10.1111/aphw.12074

49 Querstret D, Cropley M, Fife-Schaw C. The Effects of an Online Mindfulness Intervention on Perceived Stress, Depression and Anxiety in a Non-clinical Sample: A Randomised Waitlist Control Trial. *Mindfulness* 2018;**9**:1825–36. doi:10.1007/s12671-018-0925-0

50 Krusche A, Cyhlarova E, Williams JMG. Mindfulness online: An evaluation of the feasibility of a web-based mindfulness course for stress, anxiety and depression. *BMJ Open* 2013;**3**:1–10. doi:10.1136/bmjopen-2013-003498

51 Bowen DJ, Kreuter M, Spring B, *et al.* How We Design Feasibility Studies. *Am J Prev Med* 2009;**36**:452–7. doi:10.1016/j.amepre.2009.02.002

For peer review only

52 Hallingberg B, Turley R, Segrott J, *et al.* Exploratory studies to decide whether and how to proceed with full-scale evaluations of public health interventions: a systematic review of guidance. *Pilot Feasibility Stud* 2018;**4**:104. doi:10.1186/s40814-018-0290-8

53 Medical Research Council. Developing and evaluating complex interventions. 2008. www.mrc.ac.uk/complexinterventionsguidance (accessed 17 Jun 2020).

54 Chan A-W, Tetzlaff JM, Altman DG, *et al.* SPIRIT 2013 Statement: Defining Standard Protocol Items for Clinical Trials. *Ann Intern Med* 2013;**158**:200–7. doi:10.7326/0003-4819-158-3-201302050-00583

55 Kabat-Zinn J. *Full catastrophe living : how to cope with stress, pain and illness using mindfulness meditation / by Jon Kabat-Zinn.* Revised an. London : Piatkus, c2013. 2013.

56 Segal ZV, Williams JMG, Teasdale JD. *Mindfulness-based cognitive therapy for depression*. 2nd ed. New York: : Guilford Press 2013.

57 Amick BC, Lerner D, Rogers WH, *et al.* A Review of Health-Related Work Outcome Measures and Their Uses, and Recommended Measures: *Spine* 2000;**25**:3152–60. doi:10.1097/00007632-200012150-00010

58 Abma FI, van der Klink JJL, Bültmann U. The Work Role Functioning Questionnaire 2.0 (Dutch Version): Examination of its Reliability, Validity and Responsiveness in the General Working Population. *J Occup Rehabil* 2013;**23**:135–47. doi:10.1007/s10926-012-9379-8

59 Ramada JM, Serra C, Amick BC, *et al.* Reliability and Validity of the Work Role Functioning Questionnaire (Spanish Version). *J Occup Rehabil* 2014;**24**:640–9. doi:10.1007/s10926-013-9495-0

60 Johansen T, Lund T, Jensen C, *et al.* Cross-cultural adaptation of the Work Role Functioning Questionnaire 2.0 to Norwegian and Danish. *Work* 2018;**59**:471–8. doi:10.3233/WOR-182705

61 Schaufeli WB, Bakker AB. Utrecht Work Engagement Scale. Preliminary Manual. 2004.

62 Ilmarinen J. The Work Ability Index (WAI). *Occup Med* 2007;**57**:160–160. doi:10.1093/occmed/kqm008

63 Marks IM, Bird J. *Behavioural psychotherapy: Maudsley pocket book of clinical management*. 6th ed. Bristol: : Wright 1986.

64 Zahra D, Qureshi A, Henley W, *et al.* The work and social adjustment scale: Reliability, sensitivity and value. *Int J Psychiatry Clin Pract* 2014;**18**:131–8. doi:10.3109/13651501.2014.894072

65 Abma FI, Bjorner JB, Amick BC, *et al.* Two valid and reliable work role functioning questionnaire short versions were developed: WRFQ 5 and WRFQ 10. *J Clin Epidemiol* 2019;**105**:101–11. doi:10.1016/j.jclinepi.2018.09.005

For peer review only

66 Verbruggen F, Logan GD. Response inhibition in the stop-signal paradigm. *Trends Cogn Sci* 2008;**12**:418–24. doi:10.1016/j.tics.2008.07.005

67 Cools R, Clark L, Owen AM, *et al.* Defining the Neural Mechanisms of Probabilistic Reversal Learning Using Event-Related Functional Magnetic Resonance Imaging. *J Neurosci* 2002;**22**:4563–7. doi:10.1523/JNEUROSCI.22-11-04563.2002

68 Bradley MM, Lang PJ. International Affective Picture System. In: Zeigler-Hill V, Shackelford TK, eds. *Encyclopedia of Personality and Individual Differences*. Cham: : Springer International Publishing 2017. 1–4. doi:10.1007/978-3-319-28099-8_42-1

69 Tennant R, Hiller L, Fishwick R, *et al.* The Warwick-Edinburgh Mental Well-being Scale (WEMWBS): Development and UK validation. *Health Qual Life Outcomes* 2007;**5**:63. doi:10.1186/1477-7525-5-63

70 Ng Fat L, Scholes S, Boniface S, *et al.* Evaluating and establishing national norms for mental wellbeing using the short Warwick–Edinburgh Mental Well-being Scale (SWEMWBS): findings from the Health Survey for England. *Qual Life Res* 2017;**26**:1129–44. doi:10.1007/s11136-016-1454-8

71 Cohen S, Kamarck T, Mermelstein R. A Global Measure of Perceived Stress. *J Health Soc Behav* 1983;**24**:385. doi:10.2307/2136404

72 Kroenke K, Spitzer RL, Williams JBW. The PHQ-9. *J Gen Intern Med* 2001;**16**:606–13. doi:10.1046/j.1525-1497.2001.016009606.x

73 Spitzer RL, Kroenke K, Williams JBW, *et al.* A Brief Measure for Assessing Generalized Anxiety Disorder: The GAD-7. *Arch Intern Med* 2006;**166**:1092–7. doi:10.1001/archinte.166.10.1092

74 Löwe B, Decker O, Müller S, *et al.* Validation and standardization of the Generalized Anxiety Disorder Screener (GAD-7) in the general population. *Med Care* 2008;**46**:266–74. doi:10.1097/MLR.0b013e318160d093

75 Brown KW, Ryan RM. The Benefits of Being Present: Mindfulness and Its Role in Psychological Well-Being. *J Pers Soc Psychol* 2003;**84**:822–48. doi:10.1037/0022-3514.84.4.822

76 Mathieu E, McGeechan K, Barratt A, *et al.* Internet-based randomized controlled trials: a systematic review. *J Am Med Inform Assoc* 2013;**20**:568–76. doi:10.1136/amiajnl-2012-001175

77 Billingham SA, Whitehead AL, Julious SA. An audit of sample sizes for pilot and feasibility trials being undertaken in the United Kingdom registered in the United Kingdom Clinical Research Network database. *BMC Med Res Methodol* 2013;**13**:104. doi:10.1186/1471-2288-13-104

78 Harris PA, Taylor R, Thielke R, *et al.* Research electronic data capture (REDCap)--a metadata-driven methodology and workflow process for providing translational research informatics support. *J Biomed Inform* 2009;**42**:377–81. doi:10.1016/j.jbi.2008.08.010

For peer review only

79 Harris PA, Taylor R, Minor BL, *et al.* The REDCap consortium: Building an international community of software platform partners. *J Biomed Inform* 2019;**95**:103208. doi:10.1016/j.jbi.2019.103208

80 Uschner D, Schindler D, Hilgers R-D, *et al.* randomizeR: An R package for the assessment and implementation of randomization in clinical trials. *J Stat Softw* 2018;**85**:1–22. doi:10.18637/jss.v085.i08

81 Vainre M. *Randomisation for the SWELL Trial*. 2021. https://github.com/mvainre/SWELL (accessed 21 Jan 2021).

82 Baer R, Crane C, Miller E, *et al.* Doing no harm in mindfulness-based programs: Conceptual issues and empirical findings. *Clin Psychol Rev* 2019;**71**:101–14. doi:10.1016/j.cpr.2019.01.001

83 Van Dam NT, Galante J. Underestimating harm in mindfulness-based stress reduction. *Psychol Med* 2020;:1–3. doi:10.1017/S003329172000447X

84 Lange K, Kühn S, Filevich E. "Just Another Tool for Online Studies" (JATOS): An Easy Solution for Setup and Management of Web Servers Supporting Online Studies. *PLOS ONE* 2015;**10**:e0130834. doi:10.1371/journal.pone.0130834

For peer review only

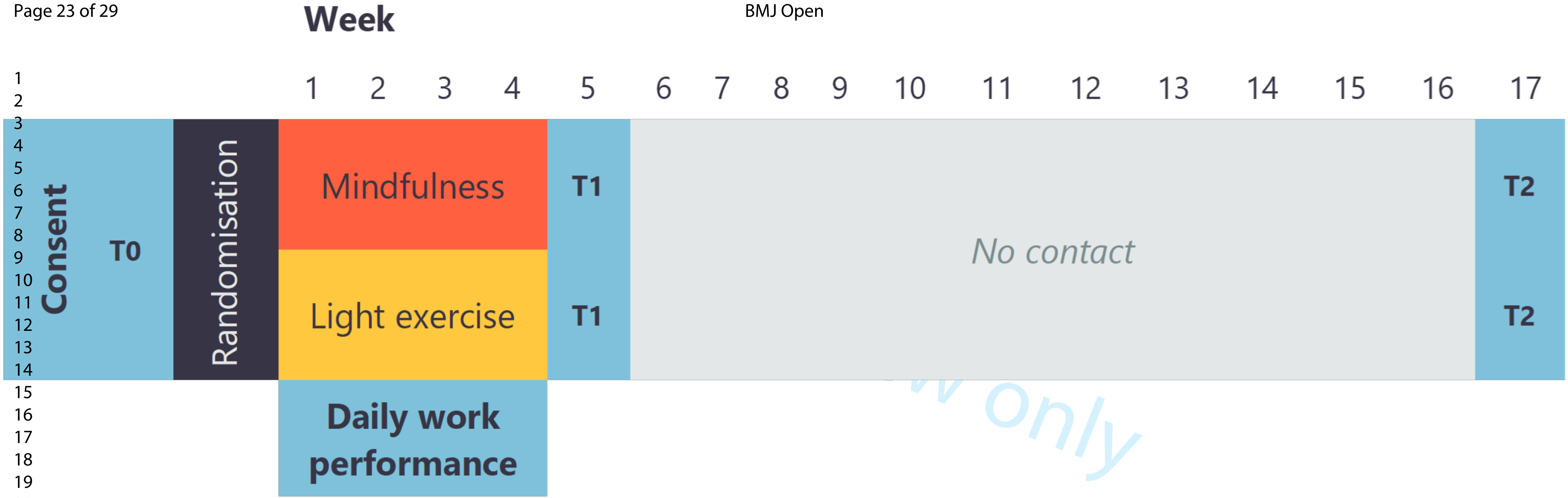


*Note*. Items **in bold** denote data collection.

STANDARD PROTOCOL ITEMS: RECOMMENDATIONS FOR INTERVENTIONAL TRIALS

SPIRIT 2013 Checklist: Recommended items to address in a clinical trial protocol and related documents*

| Section/item | ItemNo | Description | Where to find |
|---|---|---|---|
| **Administrative information** | | | |
| Title | 1 | Descriptive title identifying the study design, population, interventions, and, if applicable, trial acronym | Title page |
| Trial registration | 2a | Trial identifier and registry name. If not yet registered, name of intended registry | Abstract |
| | 2b | All items from the World Health Organization Trial Registration Data Set | Outlined in trial registration |
| Protocol version | 3 | Date and version identifier | Abstract |
| Funding | 4 | Sources and types of financial, material, and other support | Funding statement, p 15 |
| Roles and responsibilities | 5a | Names, affiliations, and roles of protocol contributors | Title page, Contributors, pp 15-16 |
| | 5b | Name and contact information for the trial sponsor | Contributors, trial registration |
| | 5c | Role of study sponsor and funders, if any, in study design; collection, management, analysis, and interpretation of data; writing of the report; and the decision to submit the report for publication, including whether they will have ultimate authority over any of these activities | Contributors, p 15 |

For peer review only

| | | | |
|---|---|---|---|
| | 5d | Composition, roles, and responsibilities of the coordinating centre, steering committee, endpoint adjudication committee, data management team, and other individuals or groups overseeing the trial, if applicable (see Item 21a for data monitoring committee) | pp 13-14 |
| **Introduction** | | | |
| Background and rationale | 6a | Description of research question and justification for undertaking the trial, including summary of relevant studies (published and unpublished) examining benefits and harms for each intervention | pp 5-6 |
| | 6b | Explanation for choice of comparators | p 7 |
| Objectives | 7 | Specific objectives or hypotheses | p 6 |
| Trial design | 8 | Description of trial design including type of trial (eg, parallel group, crossover, factorial, single group), allocation ratio, and framework (eg, superiority, equivalence, noninferiority, exploratory) | p 6 |
| **Methods: Participants, interventions, and outcomes** | | | |
| Study setting | 9 | Description of study settings (eg, community clinic, academic hospital) and list of countries where data will be collected. Reference to where list of study sites can be obtained | p 6 |
| Eligibility criteria | 10 | Inclusion and exclusion criteria for participants. If applicable, eligibility criteria for study centres and individuals who will perform the interventions (eg, surgeons, psychotherapists) | p 6 |
| Interventions | 11a | Interventions for each group with sufficient detail to allow replication, including how and when they will be administered | p 7 |
| | 11b | Criteria for discontinuing or modifying allocated interventions for a given trial participant (eg, drug dose change in response to harms, participant request, or improving/worsening disease) | p 7 and p 13 |


For peer review only - http://bmjopen.bmj.com/site/about/guidelines.xhtml

| | | | |
|---|---|---|---|
| | 11c | Strategies to improve adherence to intervention protocols, and any procedures for monitoring adherence (eg, drug tablet return, laboratory tests) | p 7 |
| | 11d | Relevant concomitant care and interventions that are permitted or prohibited during the trial | p 6 |
| Outcomes | 12 | Primary, secondary, and other outcomes, including the specific measurement variable (eg, systolic blood pressure), analysis metric (eg, change from baseline, final value, time to event), method of aggregation (eg, median, proportion), and time point for each outcome. Explanation of the clinical relevance of chosen efficacy and harm outcomes is strongly recommended | pp 8-11 |
| Participant timeline | 13 | Time schedule of enrolment, interventions (including any run-ins and washouts), assessments, and visits for participants. A schematic diagram is highly recommended (see Figure) | p 8 |
| Sample size | 14 | Estimated number of participants needed to achieve study objectives and how it was determined, including clinical and statistical assumptions supporting any sample size calculations | Sample size, p 11 |
| Recruitment | 15 | Strategies for achieving adequate participant enrolment to reach target sample size | Recruitment, p 12 |
| **Methods: Assignment of interventions (for controlled trials)** | | | |
| Allocation: | | | |
| Sequence generation | 16a | Method of generating the allocation sequence (eg, computer-generated random numbers), and list of any factors for stratification. To reduce predictability of a random sequence, details of any planned restriction (eg, blocking) should be provided in a separate document that is unavailable to those who enrol participants or assign interventions | Randomisation, p12 and GitHub |
| Allocation concealment mechanism | 16b | Mechanism of implementing the allocation sequence (eg, central telephone; sequentially numbered, opaque, sealed envelopes), describing any steps to conceal the sequence until interventions are assigned | Randomisation, p12 |

| | | | |
|---|---|---|---|
| Implementation | 16c | Who will generate the allocation sequence, who will enrol participants, and who will assign participants to interventions | Randomisation, p12 |
| Blinding (masking) | 17a | Who will be blinded after assignment to interventions (eg, trial participants, care providers, outcome assessors, data analysts), and how | Randomisation, p12 |
| | 17b | If blinded, circumstances under which unblinding is permissible, and procedure for revealing a participant's allocated intervention during the trial | Randomisation, p12 |
| **Methods: Data collection, management, and analysis** | | | |
| Data collection methods | 18a | Plans for assessment and collection of outcome, baseline, and other trial data, including any related processes to promote data quality (eg, duplicate measurements, training of assessors) and a description of study instruments (eg, questionnaires, laboratory tests) along with their reliability and validity, if known. Reference to where data collection forms can be found, if not in the protocol | p 8, Data management, p 14 |
| | 18b | Plans to promote participant retention and complete follow-up, including list of any outcome data to be collected for participants who discontinue or deviate from intervention protocols | p 12 |
| Data management | 19 | Plans for data entry, coding, security, and storage, including any related processes to promote data quality (eg, double data entry; range checks for data values). Reference to where details of data management procedures can be found, if not in the protocol | Data management, p 14 |
| Statistical methods | 20a | Statistical methods for analysing primary and secondary outcomes. Reference to where other details of the statistical analysis plan can be found, if not in the protocol | Statistical methods, p 13 |
| | 20b | Methods for any additional analyses (eg, subgroup and adjusted analyses) | Statistical methods, p 13 |
| | 20c | Definition of analysis population relating to protocol non-adherence (eg, as randomised analysis), and any statistical methods to handle missing data (eg, multiple imputation) | Statistical methods, p 13 |

**Methods: Monitoring**

| | | | |
|---|---|---|---|
| Data monitoring | 21a | Composition of data monitoring committee (DMC); summary of its role and reporting structure; statement of whether it is independent from the sponsor and competing interests; and reference to where further details about its charter can be found, if not in the protocol. Alternatively, an explanation of why a DMC is not needed | p 13 |
| | 21b | Description of any interim analyses and stopping guidelines, including who will have access to these interim results and make the final decision to terminate the trial | p 13-14 |
| Harms | 22 | Plans for collecting, assessing, reporting, and managing solicited and spontaneously reported adverse events and other unintended effects of trial interventions or trial conduct | p 13-14 |
| Auditing | 23 | Frequency and procedures for auditing trial conduct, if any, and whether the process will be independent from investigators and the sponsor | p 13-14 |

**Ethics and dissemination**

| | | | |
|---|---|---|---|
| Research ethics approval | 24 | Plans for seeking research ethics committee/institutional review board (REC/IRB) approval | p 14 |
| Protocol amendments | 25 | Plans for communicating important protocol modifications (eg, changes to eligibility criteria, outcomes, analyses) to relevant parties (eg, investigators, REC/IRBs, trial participants, trial registries, journals, regulators) | p 14 |
| Consent or assent | 26a | Who will obtain informed consent or assent from potential trial participants or authorised surrogates, and how (see Item 32) | p 14 |
| | 26b | Additional consent provisions for collection and use of participant data and biological specimens in ancillary studies, if applicable | NA |
| Confidentiality | 27 | How personal information about potential and enrolled participants will be collected, shared, and maintained in order to protect confidentiality before, during, and after the trial | p 14 |

For peer review only

| | | | |
|---|---|---|---|
| Declaration of interests | 28 | Financial and other competing interests for principal investigators for the overall trial and each study site | p 15 |
| Access to data | 29 | Statement of who will have access to the final trial dataset, and disclosure of contractual agreements that limit such access for investigators | p 14 |
| Ancillary and post-trial care | 30 | Provisions, if any, for ancillary and post-trial care, and for compensation to those who suffer harm from trial participation | p 13-14 |
| Dissemination policy | 31a | Plans for investigators and sponsor to communicate trial results to participants, healthcare professionals, the public, and other relevant groups (eg, via publication, reporting in results databases, or other data sharing arrangements), including any publication restrictions | p 14 |
| | 31b | Authorship eligibility guidelines and any intended use of professional writers | p 14 |
| | 31c | Plans, if any, for granting public access to the full protocol, participant-level dataset, and statistical code | p 14 |
| **Appendices** | | | |
| Informed consent materials | 32 | Model consent form and other related documentation given to participants and authorised surrogates | See supplementary file 2 |
| Biological specimens | 33 | Plans for collection, laboratory evaluation, and storage of biological specimens for genetic or molecular analysis in the current trial and for future use in ancillary studies, if applicable | NA |

*It is strongly recommended that this checklist be read in conjunction with the SPIRIT 2013 Explanation & Elaboration for important clarification on the items. Amendments to the protocol should be tracked and dated. The SPIRIT checklist is copyrighted by the SPIRIT Group under the Creative Commons "Attribution-NonCommercial-NoDerivs 3.0 Unported" license.


For peer review only - http://bmjopen.bmj.com/site/about/guidelines.xhtml
